# The landscape of gut microbiota in hepatocarcinogenesis: a comprehensive review of pathogenesis and therapeutic interventions

**DOI:** 10.1097/JS9.0000000000003511

**Published:** 2025-09-22

**Authors:** Zhi-Bo Yan, Cheng-Long Han, Ji-Sen Jia, Han Li, Dong-Hai Lu, Qi-Hang Cao, Yu-Xuan Wang, Ke-Fan Jiao, Qiao He, Sheng-Xuan Peng, Dao-Lin Zhang, Qiang Wang, Tao Li

**Affiliations:** Department of General Surgery, Qilu Hospital of Shandong University, Jinan, China

**Keywords:** gut microbiota, hepatocellular carcinoma, metabolic reprogramming, tumor immune microenvironment

## Abstract

Primary liver cancer (PLC) represents a significant global health burden, with hepatocellular carcinoma (HCC) being its predominant subtype. The gut microbiota plays a crucial role in the pathogenesis, treatment, and postoperative recovery of HCC through its regulatory functions along the gut–liver axis. This review systematically elucidates the role of gut microbiota dysbiosis and associated metabolites in the pathogenesis of HCC, specifically addressing the underlying mechanisms whereby gut microbiota and their metabolites mediate hepatic metabolic reprogramming, remodel the immune microenvironment, and promote HCC progression through crosstalk with intratumoral bacteria. It further explores the impact of the gut microbiota on immunotherapy, molecular targeted therapy, conventional chemotherapy, and surgical outcomes. Additionally, the review comprehensively outlines therapeutic strategies targeting the gut microbiota, including oral probiotics, antibiotics, fecal microbiota transplantation (FMT), particular small molecules, and traditional Chinese medicine. In summary, this review provides a comprehensive overview of how the gut microbiota influences the development and treatment of HCC and offers a theoretical foundation for targeting the microbiota to improve surgical prognosis in HCC patients.

**HIGHLIGHTS**

- Systematic review linking gut microbiota to hepatocellular carcinoma (HCC) via a metabolic-immune network.
- Gut microbiota affects liver glucose and lipid metabolism during the progression of HCC.
- Gut microbiota dysbiosis promotes HCC via modulating the tumor immune microenvironment.
- Systematic review of innovative therapeutic strategies targeting gut microbiota in HCC.

## Introduction

Primary liver cancer (PLC) ranks as the sixth most prevalent malignancy and the third leading cause of cancer-related deaths globally (especially among middle-aged and elderly patients)[1]. In 2020, it accounted for an estimated 905 700 new cases and 830 200 fatalities worldwide[2]. East Asia, particularly China, shoulders a substantial portion of this burden, accounting for 45.3% of incident cases and 47.1% of mortality[3]. Hepatocellular carcinoma (HCC) constitutes the predominant PLC pathological subtype in this region – comprising approximately 80% of cases – significantly exceeding the proportion of intrahepatic cholangiocarcinoma (15%) and other rare variants[2]. The gut microbiota plays a pivotal role in hepatocarcinogenesis and further progression, mediated by the bidirectional interactions of the gut-liver axis. Liver disease progression can compromise intestinal barrier integrity, resulting in the translocation of microbial metabolites and subsequent chronic liver inflammation. Conversely, targeted interventions of the microbiota, such as the administration of specific probiotic strains or fecal microbiota transplantation (FMT), can exert significant influence on the trajectory of HCC and treatment outcomes. Importantly, the gut microbiota exhibits intricate crosstalk with the metabolic reprogramming characteristic of HCC and dynamically shapes the tumor immune microenvironment. In that case, this review delves into the gut microbiota’s role in HCC pathogenesis and explores microbiota-targeted therapeutic strategies, with a particular emphasis on elucidating the mechanisms through which these microbial communities impact liver metabolic reprogramming and immune microenvironment modifications. To guarantee the transparency in the artificial intelligence reporting in this review, we followed the Transparency In The reporting of Artificial INtelligence—the TITAN guideline[4].

## Bibliometric analysis of research trends and emerging hotspots

Given the exponential growth and interdisciplinary nature of research on the gut microbiota–liver cancer axis, this study first conducted a comprehensive bibliometric analysis to objectively delineate the current research landscape. The analysis aimed to quantitatively identify evolving research frontiers, collaborative networks, and emerging priorities. Insights derived from the systematic analysis of 773 publications highlighted the significance of this field and underscored the need to integrate emerging evidence on mechanistic pathways and therapeutic innovations into a review. The bibliometric data were extracted from the Web of Science Core Collection (SCIE database), encompassing English-language articles and reviews published between January 2015 and December 2024. Search terms included variations of “gut microbiota” (e.g., “gut microbio*,” “intestinal microbio*”) and “liver cancer” (e.g., “liver cancer,” “HCC”). Literature screening adhered to a dual-independent review protocol, with discrepancies resolved by a third-party arbitrator to ensure accuracy. All analysis was performed by VOSviewer (Version 1.6.18), CiteSpace (Version 6.1 R6) and SCImago Graphica Beta (Version 1.0.18).

**Figure 1.**
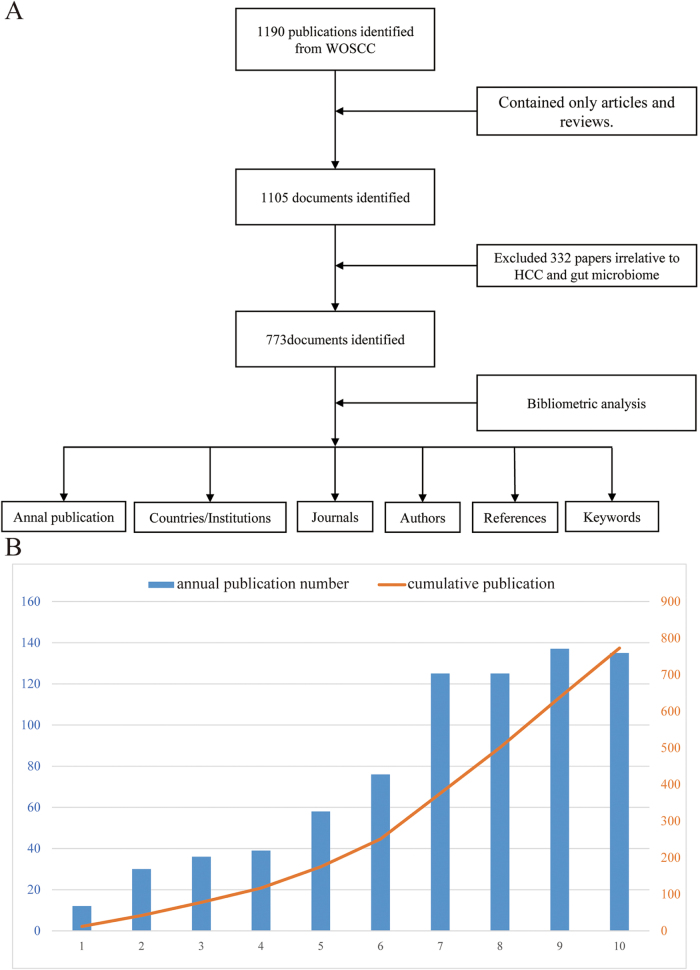
(A) The workflow diagram demonstrates the document retrieval process from the Web of Science Core Collection (WOSCC) database. (B) Annual publication output trend analysis from 2015 to 2024.

**Figure 2.**
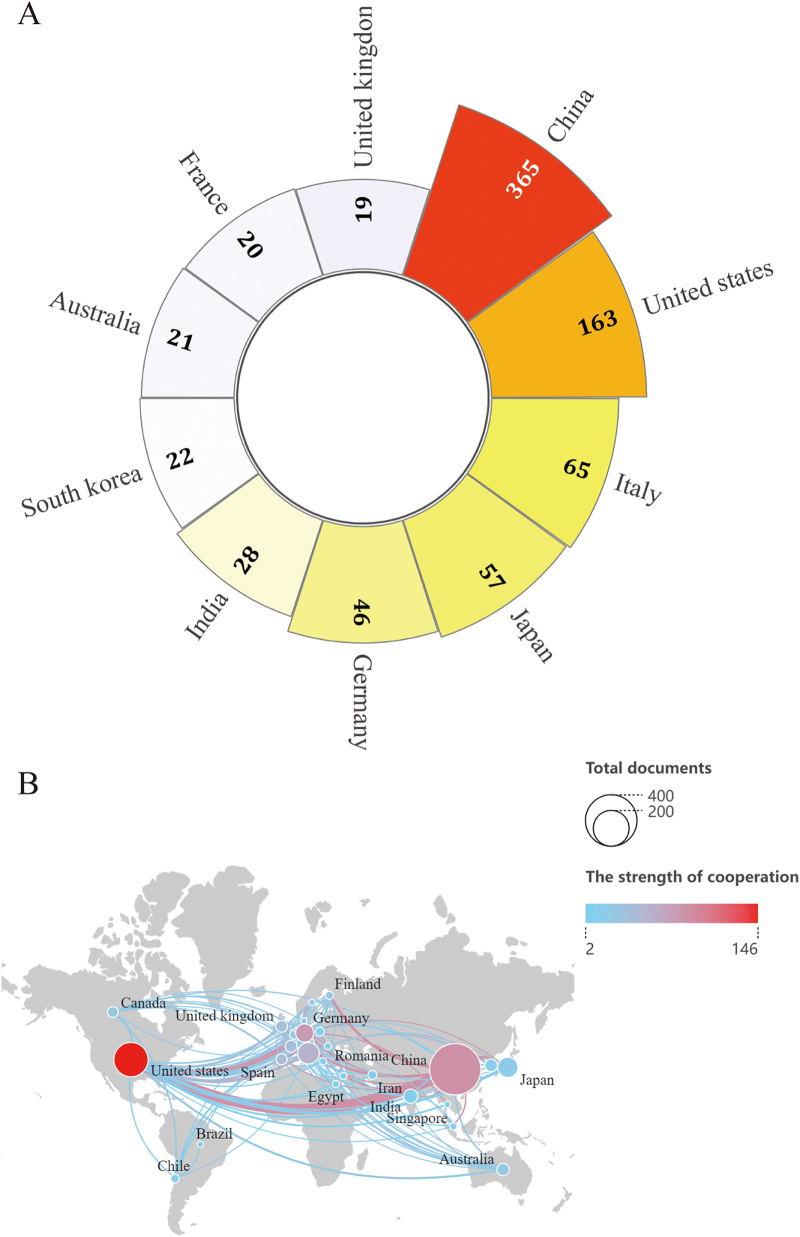
(A) Top 10 countries in terms of the number of publications. (B) The network map of countries’ cooperation (the size of the node represents the number of publications, and the color of the line shows the strength of cooperation).

**Figure 3.**
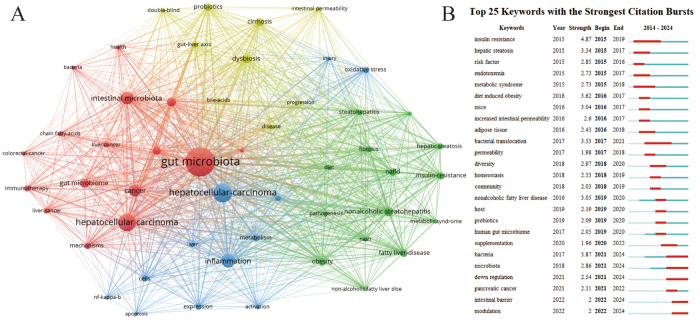
The knowledge map of active keywords (A) and the keywords with the strongest citation bursts (B) in the gut microbiota and HCC research.

The study included a total of 773 articles (389 original research articles and 384 review articles), spanning 59 countries, 1190 institutions, and 4486 authors, with 45 119 references cited (Fig. 1A). Research activity in this field has exhibited significant expansion trends, demonstrating continuous growth in annual publication volumes since 2015. The output reached its peak in 2023 at an average of 77 articles per year, while 2021 witnessed the most rapid growth with a notable year-on-year increase of 49 articles (Fig. 1B). The distribution of national contributions showed that China dominated with 351 articles (45.4%), followed by the United States (163 articles) and Italy (65 articles) (Fig. 2A). Additionally, United states was at the forefront of the international cooperation, especially with China (Fig. 2B). Among institutional outputs, Chinese institutions occupied eight of the top ten spots, with Shanghai Jiao Tong University (23 articles), Zhejiang University (21 articles), and Fudan University (21 articles) ranking in the top three (Supplementary Digital Content Figure S1, available at: http://links.lww.com/JS9/F152). However, Kumamoto University demonstrated a profound influence with a single article being cited 335.6 times. Within the core author network, Bernd Schnabl (Germany), Ki Tae Suk (South Korea), and Gang Chen (China) emerged as high-yield representatives, while the cocited author Jasmohan Singh Bajaj (United States, 394 citations) highlighted his foundational role in theory (Supplementary Digital Content Figure S2, available at: http://links.lww.com/JS9/F152). The journal analysis demonstrated that the “International Journal of Molecular Sciences” led with 34 publications and 1358 citations, but “Hepatology” exhibited academic leadership with 94 citations per article (Supplementary Digital Content Figure S3A, available at: http://links.lww.com/JS9/F152). The cocited journals were centered around “Hepatology” (3271 citations) and “Journal of Hepatology” (2159 citations), indicating a deep integration of molecular biology and clinical medicine in the interdisciplinary citation network (Supplementary Digital Content Fig. S3B, available at: http://links.lww.com/JS9/F152). Keyword clustering revealed “gut microbiota” and “hepatocellular carcinoma” as the cornerstones of the field, while the explosive growth of “intestinal barrier” and “modulation” (after 2021) signaled a shift in research toward targeted intervention strategies (Figure 3A, B). Timeline analysis further uncovered the immune system (#0 cluster) as the historical mainstay of research, with immunotherapy (#7 cluster) predicted as a future breakthrough direction, suggesting potential pathways for the translation of basic mechanisms into clinical practice (Supplementary Digital Content Figure S4, available at: http://links.lww.com/JS9/F152). In the dual map overlay, the journals housing the citing literature are represented on the left, whereas those containing the cited literature appear on the right. Citation links are denoted by curves: yellow lines illustrate the influence of molecular biology and genetics on publications in molecular biology and immunology, while green lines demonstrate the impact of the same field on medicine, medical science, and clinical publications (Supplementary Digital Content Figure S5, available at: http://links.lww.com/JS9/F152). It is worth noting that this search strategy is limited to English publications, which may miss some relevant non-English literature, thus having potential bias.

## Gut microbiota and tumor heterogeneity in hepatocellular carcinoma

Tumor heterogeneity in HCC is a major factor contributing to its high lethality rate and may dramatically influence therapeutic efficacy. HCC heterogeneity manifests at distinct levels: across the patients (interpatient heterogeneity), within the same patient (intra-patient heterogeneity), and even within the same tumor (intra-tumor heterogeneity)[5]. Emerging studies suggest a close link between the gut microbiota and HCC heterogeneity. A deeper understanding of the underlying mechanisms by which gut microbiota contribute to HCC heterogeneity is critical to improving our comprehension of HCC and optimizing patient outcomes.

For interpatient heterogeneity in HCC, the difference in the etiology of HCC appears to be a crucial factor. Research has revealed etiology-dependent differences in the gut microbiota associated with the pathogenesis of HCC[6]. Compared with nonviral HCC patients, 11 genera, that is, Erysipelotrichaceae UCG-003, *Ruminococcus gauvreauii, Faecalibacterium, Agathobacter, Coprococcus, Subdoligranulum, Lachnospiraceae* ND3007, *Lachnospiraceae* UCG-004, *Prevotella*, CAG-56, and *Holdemanella*, were significantly enriched in the viral-HCC patients, while five genera including *Ruminococcus gnavus, Bacteroides, Erysipelatoclostridium, Streptococcus*, and *Parabacteroides*, were significantly decreased[7]. One study also indicated that the *Erysipelotrichaceae* can promote the development of (HBV) Hepatitis B Virus-related HCC[8]. Notably, though belonging to the same genus, different species of *Ruminococcus* (*R. gauvreauii* and *R. gauvreauii*) present opposite trends between viral-HCC and nonviral HCC, suggesting that further studies should notice the species associated heterogeneity. Multiple short-chain fatty acids (SCFAs)-producing taxa, including *Faecalibacterium, Lachnospiraceae, Coprococcus, Agathobacter*, and *Subdoligranulum*, were less abundant in nonviral HCC compared to viral-associated HCC[7]. In agreement with that, Liu *et al*[9] also found that *Faecalibacterium* was reduced in nonviral HCC patients. Patients with metabolic dysfunction-associated steatotic liver disease associated HCC (MASLD-HCC) exhibit elevated fecal abundance of *Bacteroides ovatus, Enterococcus faecium, Kluyvera georgiana*, and *Klebsiella oxytoca*, concurrently with decreased levels of SCFAs[10]. Gut microbiota-derived SCFAs may potentiate the efficacy of immunotherapy in HCC[11]. Furthermore, the reduction in SCFAs-producing taxa like *Faecalibacterium* among MASLD patients has been also corroborated by other studies^[12,13]^, which may associated with the immunotherapy resistance in some of MASLD-HCC patients. Gut microbiota alterations are also implicated in alcoholic liver disease-associated HCC pathogenesis. *Thomasclavelia ramosa* has been identified as a significant contributor[14]. Ethanol abuse induces qualitative and quantitative changes in the intestinal flora composition, mucosal inflammation, and disruption of the intestinal barrier[15], processes that contribute to HCC development. The differential abundance of bacteria across different HCC subtypes suggests that modifying specific gut microbiota may offer therapeutic benefits for particular HCC subtypes.

The change in the pathological type of HCC is another crucial factor for interpatient heterogeneity in HCC. Clinically, cohort studies have indicated that the characteristics of tissue-resident microbiota are also linked to the pathological type of cancer^[16,17]^. According to the current World Health Organization classification, HCC encompasses several histological variants including scirrhous, macrotrabecular-massive, steatohepatitic, lymphocyte-rich, neutrophil-rich subtypes, and so on[18]. However, the differences in bacterial communities across these pathological subtypes have been insufficiently studied and merit further investigation.

Though within the same individual, significant intra-patient heterogeneity still exists (like multiple nodules may arise from distinct clones), potentially linked to the gut microbiota. One study has reported that distinct bacterial communities in HCC nodules between patients with multiple nodules arising from distinct clones (multicentric occurrence that more than one primary tumor generate simultaneously) and those with multiple nodules arising from a same clone via intrahepatic metastasis (IM)[19]. Furthermore, the epithelial–mesenchymal transition (EMT) pathway is upregulated in IM-HCC nodules and correlates with microbiota enriched in these lesions, such as *Streptococcus anginosus* and *Enterococcus faecalis*, which contribute to HCC cell migration, invasion, and disease progression in orthotopic mouse models. The intra-tumor heterogeneity in HCC may be closely associated with the interactions between different types of cell in tumor microenvironment (TME) and the microbiota-derived metabolites or intratumoral microbiota, as detailed in subsequent sections.

## Gut microbiota orchestrates metabolic and immune reprogramming in hepatocarcinogenesis: decoding the oncogenic nexus

### Gut microbiota dysbiosis promotes hepatocellular carcinoma through disruption of the intestinal mucosal barrier

Dysbiosis of the gut microbiota promotes the development and progression of HCC by disrupting the integrity of the intestinal mucosal barrier, which facilitates the translocation of lipopolysaccharide (LPS) into the liver[20]. The intestinal mucosal barrier consists of multiple layers, including epithelial cells, the mucus layer, and immune cells. Impairment of its function can lead to abnormally increased intestinal permeability, allowing bacterial metabolites such as LPS to enter the liver via the portal vein. Studies have shown that an imbalance between commensal and proinflammatory bacteria in the gut results in increased bacterial ligands and enterotoxins, further contributing to intestinal inflammation[21]. This inflammation can damage the epithelial tight junction structures (e.g., by reducing the expression of the tight junction protein ZO-1) and disrupt the integrity of the brush border, exacerbating intestinal leakage^[22–24]^. Notably, elevated circulating levels of ZO-1 are positively correlated with the progression of HCC^[23,25]^. Additionally, LPS binds to TLR4 receptors on the surface of intestinal epithelial cells, activating the TLR4/MyD88 signaling axis and upregulating the expression of myosin light chain kinase, which further weakens the intestinal barrier function^[26,27]^. It is worth noting that elevated circulating LPS levels are observed in HCC patients across multiple disease stages, suggesting a close association between intestinal leakage and HCC progression^[23,28]^.

LPS activates proinflammatory and pro-cancer signaling networks through multiple pathways upon entering the liver. First, LPS binding to TLR4 on the surface of HSCs promotes the secretion of proliferative factors, inhibits apoptosis of hepatoma cells, and enhances their survival ability[29]. HCC cells with high TLR4 expression show significantly increased sensitivity to LPS, and inhibiting this pathway can reduce the risk of tumor recurrence^[30,31]^. Second, chronic TLR4 activation positively regulates its own expression through the LIN28A/let-7 g axis, forming a positive feedback loop that continuously drives the abnormal proliferation of hepatocytes[32]. Furthermore, LPS promotes the malignant transformation of hepatic progenitor cells by inducing the secretion of IL-6 and (TNF-α) Tumor Necrosis Factor-α[33]. Recent studies have untangled that LPS can activate (EGFR) Epidermal Growth Factor Receptor signaling in (HSCs) Hepatic Stellate Cells, leading to the release of IL-8 from tumor cells and inducing angiogenesis, thereby accelerating the invasion and metastasis of HCC[34]. These mechanisms collectively explain the molecular basis for how gut microbiota dysbiosis disrupts intrahepatic homeostasis and drives HCC progression through the LPS–TLR4 axis.

### Gut microbiota-derived metabolites orchestrate hepatic metabolic reprogramming

While the effect of gut microbiota-derived metabolites on tumorigenesis has been well established, the role of those metabolites in the pathogenesis of HCC still yet to be elucidated (Supplementary Digital Content Table S1, available at: http://links.lww.com/JS9/F153). The gut microbiota functions as a sophisticated metabolic bioreactor, enzymatically processing both xenobiotic compounds and host-derived substrates to generate bioactive metabolites that critically modulate immune homeostasis. In that case, microbial dysbiosis will induce significant perturbations of those metabolites, particularly altering those involved in carcinogenesis, thereby influencing the pathogenesis of HCC. Studies elucidating the effect of gut microbiota-derived metabolites in carcinogenesis are accumulating; here, we systematically synthesize key findings regarding their mechanistic roles in bile acid (BA), lipid, glucose, and amino acid metabolism during hepatocarcinogenesis.

#### Bile acid metabolism

**Figure 4.**
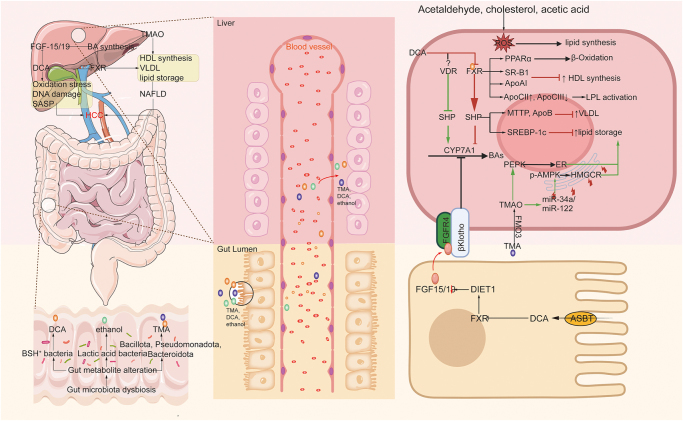
Schematic diagram illustrating how gut microbiota metabolites regulate hepatic bile acid and lipid metabolism to promote hepatocellular carcinoma (HCC) development.

BAs, as core metabolic molecules of the gut-liver axis, undergo dynamic regulation in their synthesis and metabolism by gut microbiota (Fig. 4). Primary BAs (PBAs) are synthesized in hepatocytes through cholesterol catalysis by enzymes such as CYP7A1, followed by conjugation with taurine or glycine before biliary secretion into the intestinal lumen^[35,36]^. Conjugated BAs are initially hydrolyzed by bacterial bile salt hydrolases (BSH) produced by gut microbiota, resulting in deconjugated PBAs [e.g., cholic acid (CA) and chenodeoxycholic acid (CDCA)][36]. Subsequently, a portion of PBAs undergo 7α-dehydroxylation mediated by 7α-dehydroxylase expressed in Gram-positive bacteria (*Clostridium* spp.), converting them into secondary BAs (SBAs) [deoxycholic acid (DCA) and lithocholic acid (LCA)]^[36,37]^. Approximately 95% of SBAs are efficiently reabsorbed via the enterohepatic circulation through portal venous return to hepatocytes, while the residual fraction enters systemic circulation. These circulating BAs establish negative feedback regulatory circuits through activation of farnesoid X receptor (FXR) and C, precisely regulating BA homeostasis^[20,38,39]^. Patients with metabolic dysfunction-associated steatotic liver disease/steatohepatitis (MASLD/MASH) exhibit characteristic BA dysregulation, manifested by significantly elevated total BAs levels in serum and feces, along with abnormal increases in PBAs (CA and CDCA) and conjugated BA fractions[40].

Research has identified the ileal FXR-FGF19 axis as the primary regulator of BA homeostasis: Intestinal FXR activation stimulates fibroblast growth factor (FGF) 15/19 expression, after which the protein translocates to the liver. The FGF19 protein binds hepatocyte surface receptors and potently inhibits CYP7A1 transcription – the rate-limiting enzyme in BA biosynthesis – through downstream signaling. In MASLD, this regulatory pathway becomes compromised, with observed hepatic CYP7A1 overexpression and diminished circulating FGF19 levels reflecting disruption of the canonical FXR-FGF19-CYP7A1 feedback mechanism^[35,41,42]^. BAs directly activate FXR on hepatocytes, inhibiting CYP7A1 expression via the small heterodimer partner (SHP)-dependent pathway[35]. Additionally, they stimulate the SHP-dependent vitamin D receptor, which enhances CYP7A1 expression and increases BA synthesis[43]. Notably, MASH patients demonstrate increased proportions of DCA and decreased CDCA in gut microbiota. DCA acts as an FXR antagonist in the presence of CDCA, disrupting signal transduction and exacerbating pathological BA accumulation^[42,44]^. Animal models demonstrate that DCA promotes hepatocarcinogenesis in high-fat diet-induced HCC through reactive oxygen species-mediated DNA damage[45]. Vancomycin intervention reduced the abundance of BSH-positive microbiota and decreased conjugated deoxycholic acid levels (GDCA (Glycodeoxycholic Acid) and TDCA (Taurodeoxycholic Acid)), accelerating tumor progression. This effect may stem from *Bifidobacterium* depletion, a beneficial probiotic genus in humans[46]. BA exhibits dual roles in HCC. Elevated 3-oxocholic acid and isolithocholic acid levels correlate with favorable immunotherapy outcomes, whereas DCA promotes HCC progression by inducing an immunosuppressive TME[20]. Future research should clarify how specific BAs contribute to HCC pathogenesis.

#### Lipid metabolism

The gut microbiota and its metabolites regulate the reprogramming of hepatic lipid metabolism through various mechanisms, playing a crucial role in the progression of MASLD (Fig. 4). Microbially produced endogenous ethanol disrupts intestinal barrier function, increasing toxin influx into the liver, and its metabolites, acetaldehyde and acetate, directly foster the generation of reactive oxygen species and lipid synthesis[47]. Microbiota-derived SBAs DCA suppress FXR signaling, thereby downregulating SHP expression[42]. SHP not only feedback-inhibits BA synthesis and uptake but also suppresses sterol regulatory element-binding protein-1c (SREBP-1c), a key transcription factor for *de novo* lipogenesis[48], markedly reducing hepatic lipid accumulation. In humans, FXR activation additionally upregulates peroxisome proliferator-activated receptor α, enhancing fatty acid oxidation and lowering serum triglycerides[35]. SHP further impedes hepatic very-low-density lipoprotein (VLDL) assembly and secretion by inhibiting microsomal triglyceride transfer protein and apolipoprotein B. FXR also enhances lipoprotein lipase activity by inducing its activator APOCII while repressing its inhibitor APOCIII, thereby accelerating VLDL catabolism to boost peripheral fatty acid uptake[35]. Furthermore, FXR upregulates scavenger receptor class B type 1, facilitating hepatic HDL cholesterol uptake while suppressing ApoA1, collectively fine-tuning systemic lipid distribution[35]. Elevated DCA levels promote lipid accumulation, increase serum VLDL, and drive MASLD progression, potentially serving as a key trigger for HCC development.

**Figure 5.**
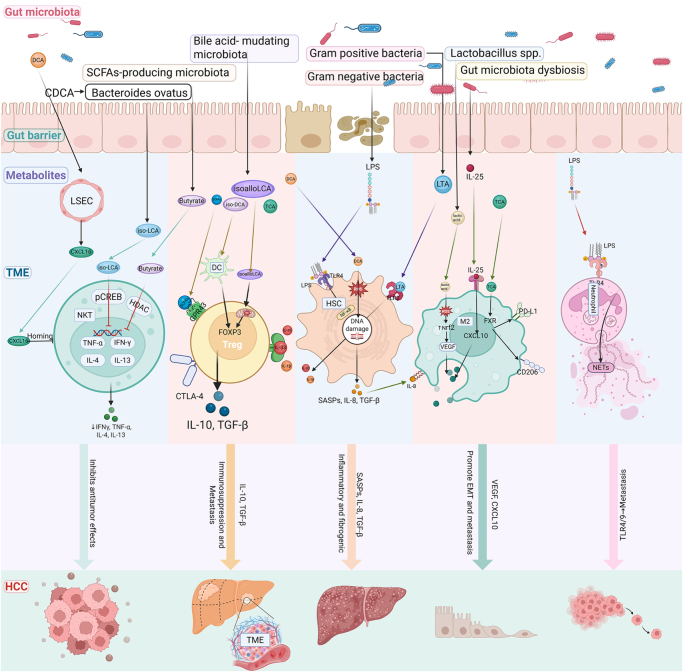
Gut microbiota and their metabolites reshape the hepatic immune microenvironment to promote HCC progression.

Notably, gut microbiota metabolize choline into trimethylamine (TMA), which enters the liver and is oxidized to trimethylamine N-oxide (TMAO) by flavin-containing monooxygenase 3 (FMO3)[49]. FMO3 knockdown reduces hepatic cholesterol, attenuates *de novo* lipogenesis mediated by liver X receptor target genes, and mitigates endoplasmic reticulum stress[50], whereas its overexpression elevates lipogenic enzyme activity and gluconeogenesis, exacerbating lipid accumulation[51]. Additionally, TMA further modulates this metabolic axis by activating FMO3. Molecular mechanistic studies reveal that TMAO triggers lipid accumulation by activating the PERK pathway in the liver ER (Endoplasmic Reticulum), a mechanism confirmed in both zebrafish and *in vitro* cell models (Fig. 5)[52]. Transcriptome analysis also indicates that TMAO treatment notably upregulates keratin 17 expression, with its level correlating positively with the extent of lipid deposition (Fig. 5)[53]. Recent investigations have shown that TMAO targets and suppresses the SIRT1 gene by elevating miR-34a and miR-122 expression, resulting in AMPK (Adenosine MonoPhosphate-activated Protein Kinase) dephosphorylation and enhanced HMG-CoA (3-Hydroxy-3- methylglutaryl-Coenzyme A) reductase activity, and thus driving cholesterol buildup and lipid metabolism reprogramming (Fig. 5)^[54,55]^. Critically, tryptamine and indole-3-acetic acid (IAA), key tryptophan metabolites, suppress hepatic lipid accumulation by inhibiting SREBP-1c/FASN signaling. However, gut microbiota dysbiosis disrupts tryptophan catabolism, reducing circulating tryptamine and IAA concentrations. This metabolic impairment diminishes their inhibitory effects on lipogenesis, thereby exacerbating MAFLD progression[56]. These findings demonstrate that gut microbiota-derived metabolites regulate hepatic lipid homeostasis by coordinately modulating oxidative stress, nuclear receptor signaling, ER dynamics, and epigenetic reprogramming. This mechanistic understanding offers new opportunities for designing multitargeted interventions against fatty liver diseases.

#### Glucose metabolism

The Warburg effect constitutes a hallmark metabolic feature of HCC, characterized by tumor cells preferentially utilizing glycolysis over oxidative phosphorylation for rapid glucose consumption and lactate production under aerobic conditions[57]. In HCC, concomitant hyperactive glycolysis and suppressed gluconeogenesis induce microenvironmental glucose deprivation. This metabolic reprogramming tightly correlates with EMT, immunosuppressive lymphocyte infiltration, and β-catenin signaling activation, collectively driving tumor progression^[58–61]^. While direct evidence linking gut microbial metabolites to HCC glucose metabolism remains limited, their indirect modulation of tumor metabolic niches may be mediated through influencing HCC risk factors, including obesity, type 2 diabetes, and MASLD. Metabolomic profiling reveals obesity-associated microbial signatures correlated with aberrant levels of glycolysis and tricarboxylic acid cycle intermediates, suggesting microbiota-mediated regulation of host glucose metabolic networks^[62,63]^. Moreover, immunotherapy responder-enriched *Ruminococcus* has also been found to have a positive relationship with serum galactaric acid[64], and disturbances in glucose metabolism were also found in mice exposed to antibiotics in early life. The early-life antibiotic exposure will accelerate the progression of MASLD-HCC[65]. SCFAs, microbial fermentation byproducts, enhance glycolysis, oxidative phosphorylation, and fatty acid synthesis. Their portal venous delivery to the liver may potentiate glycolytic flux in HCC[66]. Furthermore, microbiota-derived serotonin promotes HCC cell proliferation under nutrient deprivation, potentially via glycolytic pathway activation[67]. Notably, high-fructose diets drive hepatic *de novo* lipogenesis and glycolysis through microbial conversion to acetate, exacerbating HCC malignancy[68]. Future investigations should elucidate how microbial metabolites remodel tumor glucose metabolism by regulating key enzymes (e.g., hexokinase and lactate dehydrogenase) or signaling cascades while exploring microbiota-metabolism axis-targeted strategies for HCC intervention.

#### Amino acid metabolism

Emerging evidence suggests potential crosstalk between hepatocellular amino acid metabolic reprogramming and gut microbiota. *Candida albicans* colonization elevates plasma phenylalanine metabolite levels, while high-fructose diet-induced microbial acetate promotes HCC progression by enhancing glutaminolysis and O-GlcNAcylation^[69,70]^. Moreover, gut flora depletion can also promote liver tumorigenesis by impairing gut tryptophan metabolism and up-regulating SREBP2, which can be reversed by the supplementation with *Lactobacillus reuteri* that can produce tryptophan metabolites[71]. The kynurenine pathway has been proposed to be one of the key mechanisms used by the tumor cells to escape immune surveillance for proliferation and metastasis[72]. However, mechanistic insights into microbiota-mediated regulation of hepatic amino acid metabolism remain insufficiently substantiated. Particularly, how specific bacterial taxa and their metabolites modulate critical pathways – including glutaminolysis and urea cycle – warrants further investigation to delineate their pathophysiological roles in HCC.

### Gut microbiota-derived metabolites modulate the immune microenvironment.

Gut dysbiosis drives HCC progression by modulating immune cell phenotypes and metabolite secretion, thereby reshaping the tumor immune microenvironment. This section focuses on the mechanistic interplay between gut microbiota/metabolites and hepatic immune cell functionality, elucidating their pivotal roles in the transition from MASLD to MASH and subsequent HCC pathogenesis (Fig. 5).

#### Macrophages

Macrophages play a pivotal role in maintaining immune homeostasis, and their polarization states dynamically evolve with the progression of liver diseases. Notably, the Marco + immunosuppressive macrophage subpopulation present in the periportal area exerts its immunosuppressive function through the scavenger receptor Marco, which is dependent on gut microbiota (such as *Odoribacteraceae*) and their metabolite isolithocholic acid. Dysregulation of this system can promote the progression of inflammatory liver diseases such as primary sclerosing cholangitis and MASH[73]. In the stages of MASLD and MASH, liver macrophages are predominantly polarized toward the pro-inflammatory M1 phenotype, whereas in HCC, they shift toward the immunosuppressive M2 phenotype. The latter drives tumor progression by inhibiting apoptosis, promoting EMT, and establishing an immunosuppressive TEM^[74–77]^. Sirtuin 5 (SIRT5), a key metabolic regulator, is markedly downregulated in HCC. This deficiency impairs BA homeostasis via peroxisomal succinylation modifications, leading to taurocholic acid (TCA) accumulation. Elevated TCA levels activate nuclear receptors, inducing IL-4-dependent M2 macrophage polarization. The resulting upregulation of PD-L1 and CD206 establishes a TME that promotes tumor-initiating cell survival[78]. The gut microbiota and its metabolites modulate macrophage phenotypes through portal vein translocation. Intestinal tuft cells increase interleukin-25 (IL-25) secretion under vancomycin-induced dysbiosis. IL-25 stabilizes M2 macrophages through alternative activation while stimulating their CXCL10 production, which subsequently drives EMT in HCC[79]. Lactate derived from hepatoma cells via aerobic glycolysis markedly promotes M2 macrophage polarization through ROS-dependent Nrf2 activation and cytokine reprogramming. This lactate-mediated effect simultaneously increases VEGF expression, facilitating HCC metastasis[80]. Notably, gut microbiota (e.g., *Lactobacillus* spp.) can catabolize lactate, suggesting that exogenous lactate derived from gut microbial metabolism may contribute to M2 polarization, though further investigation is required to validate this mechanism *in vivo*. In MASH, DCA induces HSC senescence while elevating IL-8 and TGF-β (Transforming Growth Factor-β) secretion, creating a pro-inflammatory, tumor-permissive environment that activates M2 macrophages and drives HCC invasion through EMT^[81,82]^. Collectively, these mechanisms reveal how local metabolic reprogramming in the liver and gut microbiota metabolites regulate macrophage phenotypes through immune signaling networks, thereby shaping the molecular basis of the immunosuppressive microenvironment in HCC.

#### T cells

T cells in the TME maintain immune surveillance and tolerance through dynamic equilibria, with the interactions of cytotoxic CD8+ T cells, Tregs, γδT cells, and natural killer T cells (NKTs) collectively shaping the immune microenvironment of HCC. As the core effector cells of antitumor immunity, CD8+ T cells secrete INF-γ and IL-33 to inhibit the progression of HCC. However, their function is susceptible to exhaustion caused by the high expression of inhibitory receptors such as PD-1/PD-L1 and CTLA-4^[83–85]^. Intestinal dysbiosis compromises the intestinal mucosal barrier, elevating intestinal permeability and enabling microbial metabolites – particularly SBAs, LPS, and SCFAs – to translocate to the liver. These metabolites induce immunosuppression through distinct pathways: SCFAs stimulate GPR41/GPR43 signaling to directly drive Treg differentiation while enhancing IL-10 and CTLA-4 production[86]. Concurrently, LPS activates macrophages and MDSCs via TLR4, creating an immunosuppressive niche that amplifies Treg populations^[29,87]^; SBAs regulate immunity bidirectionally – isoalloLCA derivatives promote Treg differentiation by increasing FOXP3 expression through mitochondrial ROS[88], whereas isoDCA enhances Foxp3 protein levels by dampening dendritic cell immunostimulation[89]. Dysbiosis also directly expands MDSCs via TLR4 signaling, impairing CD8+ T cell function. Together, these interconnected mechanisms establish a systemic immunosuppressive state[90].

NKT cells are activated by recognizing lipid antigens derived from the gut microbiota, which are presented by CD1d molecules. Upon activation, these cells secrete cytokines such as IFN-γ, IL-4, and IL-17A. However, excessive activation of NKT cells can trigger inflammatory responses that may promote the progression of HCC by recruiting immunosuppressive cell populations^[91–94]^. Further studies have revealed that gut microbiota metabolites regulate the liver’s immune microenvironment through multiple dimensions. Mechanistic studies have shown that the deletion of the AKR1D1 gene can induce gut microbiota dysbiosis, leading to an increased proportion of *B. ovatus*. This, in turn, catalyzes the conversion of CDCA to iso-LCA. The accumulated iso-LCA directly inhibits the cytotoxic function of natural killer (NK) cells by inhibiting the phosphorylated CREB1 signaling pathway[95]. Additionally, SCFAs impair the antitumor killing ability of NKT cells by inhibiting the activity of histone deacetylase[96], while SBAs (such as DCA) can downregulate the expression of CXCL-16 in liver sinusoidal endothelial cells, thereby inhibiting the homing of NKT cells to the liver[97]. These findings systematically elucidate the key molecular pathways through which the gut microbiota, via a metabolite-immune signaling interaction network, coordinates the functions of innate immune cells (such as NK cells) and adaptive immune cell subsets (such as NKT cells), ultimately driving the pathological process of immune evasion in HCC.

#### Neutrophil

Tumor-associated neutrophils (TANs) undergo dynamic shifts in HCC, transitioning from the N1 antitumor phenotype to the N2 immunosuppressive phenotype. The ratio of TANs to lymphocytes is closely correlated with prognosis^[98–101]^. Disruptions in gut microbiota can influence the immune response by modulating circulating and intrahepatic neutrophil levels[102]. Gut-derived LPS stimulates TLR4, promoting the formation of NETs, which facilitate the transition from alcoholic steatosis to HCC[103]. Furthermore, NETs internalize into captured HCC cells and activate the TLR4/9-COX2 signaling pathway, inducing metastatic potential in hepatoma cells[104]. Although the direct regulatory mechanism of gut microbiota on neutrophil phenotypes remains to be elucidated, its indirect influence on TAN phenotypes through maintaining barrier integrity and metabolic homeostasis is becoming apparent[105].

#### Hepatic stellate cell

HSCs, as the core effector cells of liver fibrosis, shape the tumor immune microenvironment by secreting cytokines and chemokines. DCA induces HSC senescence and releases senescence-associated secretory phenotype (SASP) factors, forming a tumor-promoting microenvironment[45]. Lipoteichoic acid cooperates with obesity-induced DCA to amplify SASP in HSCs, enhancing SASP factor and COX2 expression via toll-like receptor 2 signaling[106]. Additionally, LTA triggers the release of IL-33 and IL-1β from HSCs, promoting the development of HCC by enhancing Treg activity[107]. On the other hand, LPS activates the TLR4 receptor on HSCs, triggering the NF-κB inflammatory pathway, which drives liver fibrosis and chronic inflammation, facilitating the progression of MASH-related HCC^[108,109]^. These mechanisms collectively untangle the critical role of HSCs as a metabolic-immune cross-regulatory node, suggesting that targeting HSC-mediated inflammatory fibrosis cascades and microbial metabolic imbalances may represent a novel strategy to intervene in immune evasion in HCC.

### Synergistic promotion of HCC by gut microbiota and intratumoral microbiota

Recent studies have revealed the presence of intratumoral microbiota in HCC and their potential clinical significance. These microbial populations primarily localize within the cytoplasm of both tumor and immune cells in the TME[110]. The origins of HCC-associated intratumoral microbiota appear diverse, possibly originating from the hepatic circulation, biliary tract, or peritumoral tissues[111]. During HCC progression, gut dysbiosis impairs intestinal barrier integrity, enabling gut-derived microbes and their components to enter systemic circulation through the portal venous system. This mechanism may facilitate microbial colonization of hepatic parenchyma and subsequent integration into the intratumoral microbiome^[112–114]^. Support for the gut–tumor microbial axis hypothesis comes from conserved microbial signatures between gut and tumor microbiota in HCC patients. Comparative analyses consistently show distinct enrichment of specific bacterial phyla in HCC tissues relative to normal or adjacent tissue. Huang *et al*[111] observed higher relative abundances of Proteobacteria, Firmicutes, and Actinobacteriota at the phylum level in HCC samples, with Gammaproteobacteria displaying class-level specificity. He *et al*[115] further identified elevated levels of Enterobacteriaceae, Fusobacterium, and Neisseria in HCC tumor tissues. Notably, parallel dysbiotic patterns emerge in both gut and tumor microbiota. Actinobacteriota, which expands in the gut microbiome of HCC patients[116], also shows increased abundance in tumor tissues[117]. Similarly, taxa elevated in HCC gut microbiomes (e.g., Proteobacteria[118], Firmicutes[116], Enterobacteriaceae[119], and *Lactobacillus*[120]) exhibit corresponding enrichment in matched tumor tissues^[111,115]^, while depleted gut taxa (e.g., *Faecalibacterium*[121], *Agathobacter*[118], and Ruminococcaceae[122]) demonstrate reduced tumor abundance compared to adjacent tissue[115]. These conserved dysbiosis patterns suggest potential microbial translocation or crosstalk between gut and intratumoral microbiomes in HCC.

Gut and intratumoral microbiota also share clinical risk associations. Elevated gut Proteobacteria correlate with HCC microvascular invasion (MVI)[123], intestinal inflammation/dysbiosis, and poor prognosis[124], while its tumor enrichment associates with increased AST (Aspartate Aminotransferase), ALT (Alanine Aminotransferase), and TBA levels, reflecting hepatic dysfunction[117]. Diminished gut Akkermansia links to immune dysregulation, hepatic inflammation, and IM[125], with its absence in tumors similarly predicting poorer postoperative outcomes[117]. Similarly, gut Enterobacteriaceae enrichment associates with HCC-MVI and advanced disease[123], while its tumor abundance correlates with intralesional progression and prognosis[115]. These observations suggest that gut microbes may translocate across the compromised intestinal barrier into the portal system, ultimately infiltrating the liver and contributing to intratumoral microbiota formation. As previously noted, gut microbiota may also shape the TME through immune modulation and metabolic reprogramming, potentially further influencing intratumoral microbial communities.

**Figure 6.**
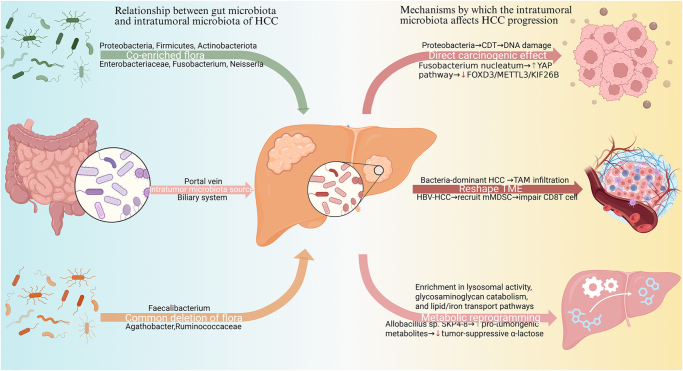
Hepatic translocation of gut microbiota as the origin of intratumoral microbiota and its mechanism in promoting HCC progression. Areas highlighted in green represent microbial taxa enriched both in the gut and within HCC tumors, while orange areas indicate taxa depleted in both locations.

The mechanisms through which intratumoral microbiota influence HCC progression remain incompletely understood, but evidence from other cancers suggests several plausible pathways. Proteobacteria are significantly enriched in HCC tumor tissues and may promote carcinogenesis by producing cytolethal distending toxin, which induces DNA damage and cellular injury in hepatocytes[126]. Elevated levels of *Fusobacterium nucleatum* in HCC[115], could similarly drive tumor progression, as this bacterium activates the YAP (Yes1 associated transcriptional regulator) pathway and suppresses the FOXD3/METTL3/KIF26B axis in colorectal cancer—a mechanism that may also operate in HCC[127], beyond direct oncogenic effects, intratumoral microbiota actively reshape the TME by modulating immune function and metabolism. Li *et al’s*[128] classification of HBV-HCC patients based on microbial profiles revealed that the bacteria-dominant subtype correlated with larger tumors, greater invasiveness, reduced survival, and increased M2 macrophage infiltration compared to the virus-dominant subtype. HBV appears to create a permissive niche for specific microbes, which in turn recruit immunosuppressive myeloid-derived suppressor cells (mMDSC and pmnMDSC) while impairing CD8+ T cell activity, collectively dampening antitumor immunity and accelerating disease progression[129]. Metabolic reprogramming represents another mechanism through which intratumoral bacteria may influence HCC, though the underlying pathways require further investigation. Functional analyses reveal microbial gene enrichment in lysosomal activity, glycosaminoglycan catabolism, and lipid/iron transport pathways in tumors relative to normal tissue. Distinct metabolic shifts – such as increased myristic acid and citrulline alongside decreased lactobionic acid – closely align with variations in bacterial abundance. *Allobacillus* sp. SKP4-8 shows a positive association with pro-tumorigenic metabolites but inversely correlates with tumor-suppressive α-lactose, whereas *Pseudomonas* spp. display the opposite pattern[130]. These observations suggest that intratumoral microbes actively reshape the metabolic landscape of the TME, potentially contributing to oncogenesis through metabolite-mediated mechanisms (Fig. 6)[131].

## Mechanisms underlying the impact of gut microbiota on hepatocellular carcinoma therapy

**Figure 7.**
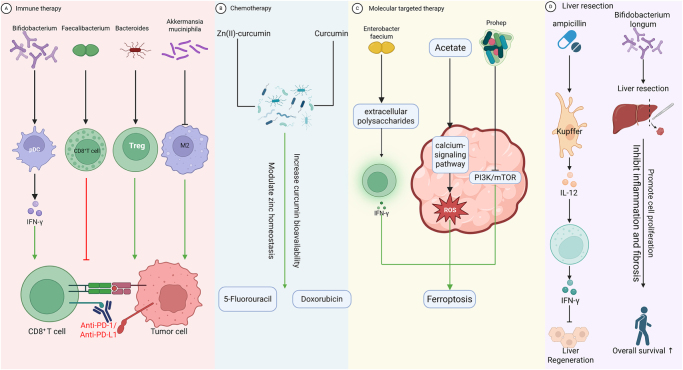
Interactions of the gut microbiota with therapeutics in HCC: Immunotherapy, chemotherapy, molecular targeted therapy, and surgery. Antagonistic effects are depicted in red; synergistic effects are depicted in green.

The contemporary therapeutic landscape for HCC has been revolutionized by groundbreaking advances in immune checkpoint inhibitors (ICIs), molecular targeted therapy, and optimized chemotherapy regimens, offering durable clinical benefits to a considerable HCC patient population (Fig. 7). Nevertheless, a substantial proportion of HCC cases still exhibit suboptimal therapeutic responses, with growing evidence implicating gut microbiota dysbiosis as a key modulator of treatment efficacy through multifaceted host–microbe interactions. This section systematically examines the microbiota-mediated mechanisms influencing the therapy efficacy of ICIs, molecular targeted therapy, and chemotherapy.

### Gut microbiota modulates immune checkpoint inhibitor therapy

Advanced HCC, ranked as the sixth most lethal malignant tumor globally, faces a significant clinical challenge in terms of ICIs resistance, even though ICIs provide benefit to numerous patients[132]. ICIs activate T-cell antitumor effects by targeting pathways such as CTLA-4 and PD-1/PD-L1, yet their efficacy is significantly modulated by the TME and host factors^[133–135]^. Recent studies have revealed that gut microbiota profoundly influence ICIs’ sensitivity through metabolic products and immune regulatory networks, exhibiting unique mechanisms in HCC. Early research untangled that *Bifidobacterium* enhances the efficacy of PD-1 inhibitors by promoting dendritic cell maturation and IFN-γ secretion, and this effect can be transferred across strains via FMT[136]. A pivotal 2018 study found that patients enriched with Clostridiales and Ruminococcaceae showed significantly improved response rates to PD-1 blockade therapy[137]. Specifically, the abundance of *Faecalibacterium* (belonging to Ruminococcaceae) positively correlated with tumor-infiltrating CD8+ T-cell levels and was associated with longer progression-free survival (PFS)[137]. Conversely, patients enriched with *Bacteroides* exhibited expansion of peripheral Tregs and MDSCs, indicating the formation of a microbiota-mediated immunosuppressive microenvironment[137]. Notably, HCC-specific microbiota characteristics are gradually emerging: A 2021 study discovered that patients enriched with *Lachnospiraceae* GAM79 and *Alistipes* sp. Marseille-P5997 had significantly prolonged overall survival (OS) after PD-1/PD-L1 treatment, while the high abundance of *Veillonellaceae* predicted poor prognosis[138]. A 2022 study emphasized that an increased abundance of *Lachnoclostridium* and *Lachnospiraceae*, combined with the depletion of Prevotella 9, significantly improved ICI treatment response. Although patients enriched with *Veillonella* had a higher response rate to ICIs, their prognosis was still influenced by the complexity of microbiota interactions[139]. Further multikingdom microbiome analysis revealed significant enrichment of *Phascolarctobacterium faecium* and *Cladosporium fungi* in the durable clinical benefit group. This group’s microbiota phylogeny was dominated by *Candidatus* and Ascomycota, with significantly higher metabolite abundance and complexity of microbiota–metabolite interaction networks compared to the nonbenefit group (NDB)[140]. In contrast, the overproliferation of Prevotella 9, *Actinomyces* sp. ICM47 and *Basidiomycota* fungi in the NDB group may reduce treatment response by reshaping the immunosuppressive microenvironment[140]. Recent studies indicate that *Akkermansia muciniphila* suppresses mMDSC and M2 macrophage activity while enhancing T-cell responses, thereby improving the efficacy of PD-1/PD-L1 therapy[141]. This evidence suggests that gut microbiota dynamically regulates HCC immunotherapy response through cross-kingdom bacterial–fungal interactions and metabolic reprogramming, providing a theoretical basis for precision microbiota intervention strategies.

### Gut microbiota modulates chemotherapeutic efficacy

Chemotherapy induces tumor cell death through chemical drugs, but its efficacy is limited in advanced HCC, primarily due to treatment toxicity, difficulties in dose regulation, and tumor heterogeneity^[142–145]^. Recent studies have focused on the regulatory role of gut microbiota in chemotherapy efficacy, revealing its enhancement of drug sensitivity through metabolic intervention and immune modulation. Clinical evidence suggests a significant association between gut microbiota dysbiosis after transarterial chemoembolization and increased risk of HCC recurrence, highlighting the importance of microbiota homeostasis in chemotherapy prognosis[143]. The use of carbapenem antibiotics can disrupt microbiota composition, leading to shortened PFS and OS in patients, and antianerobic antibiotics have been identified as independent risk factors for poor prognosis, emphasizing the criticality of microbiota management during chemotherapy[143]. Curcumin, as a chemotherapy sensitizer, enhances the efficacy of doxorubicin and 5-fluorouracil through gut microbiota-mediated zinc homeostasis regulation. The sensitization effect disappears after microbiota depletion, confirming the central role of microbiota in drug metabolism^[144,145]^. Mechanistic studies have shown that curcumin reverses chemotherapy resistance by modulating specific microbiota functions to reshape the host metabolic microenvironment[144]. These findings provide a theoretical basis for individualized chemotherapy strategies based on microbiota regulation, and further exploration of microbiota–drug interaction networks is needed to optimize chemotherapy regimens and improve patient survival outcomes.

### Gut microbiota enhances molecular targeted therapy efficacy

Sorafenib, as a first-line targeted drug for HCC[146], has its efficacy limited by tumor heterogeneity and resistance mechanisms. Studies have revealed that *Enterobacter faecium* stimulates the secretion of interferon-γ by CD8+ T cells through extracellular polysaccharides, synergistically inducing ferroptosis in hepatoma cells with sorafenib, thereby significantly enhancing the antitumor effect[147]. Furthermore, acetate, a metabolite of gut microbiota, can amplify sorafenib’s tumor-suppressing action by activating calcium-signaling pathways and promoting the accumulation of reactive oxygen species[148]. Another study demonstrated that Prohep, a probiotic mixture, enhanced sorafenib efficacy by activating MAPK (Mitogen-Activated Protein Kinase) while inhibiting PI3K/mTOR, concurrently elevating butyrate production[149]. These evidences suggest that gut microbiota and their metabolites, through the modulation of immune responses and oxidative stress pathways, have the potential to reverse targeted therapy resistance and improve drug sensitivity, laying a molecular foundation for the development of microbiota-targeted combination therapy strategies.

### Gut microbiota improves liver resection prognosis

Hepatectomy remains the standard treatment for early-stage HCC[150], typically yielding favorable outcomes. Emerging evidence indicates that the gut microbiota influences postoperative recovery after liver resection. Particular genera like *Bifidobacterium* and *Lactobacillus* actively modulate liver injury repair and regeneration[151], with specific *Bifidobacterium* strains enhancing hepatoprotection through IL-10 induction[152]. In murine models, ampicillin-induced depletion of commensal microbiota impairs liver regeneration after partial hepatectomy. This effect stems from Kupffer cell-derived IL-12 promoting excessive activation of hepatic NKTs, which subsequently inhibits liver regeneration via IFN-γ signaling[153]. The pace of liver function recovery postoperatively significantly impacts HCC prognosis. Administration of *Bifidobacterium longum* has been shown to accelerate functional recovery and improve outcomes, including 1-year OS[154]. Intriguingly, FMT or gut *Streptococcus* enrichment in neoadjuvant chemoimmunotherapy responders may augment anti-PD-1 efficacy by boosting intratumoral *Streptococcus* and expanding tumor-infiltrating CD8^+^ T lymphocytes, explaining the association between *Streptococcus* abundance and extended PFS in esophageal squamous cell carcinoma trials[155]. Collectively, these findings highlight the gut microbiota’s dual role in posthepatectomy recovery and neoadjuvant therapy potentiation. Nevertheless, the mechanistic basis for its prognostic impact in multimodal neoadjuvant chemoimmunotherapy-surgery regimens for HCC requires deeper exploration.

### Gut microbiota enhances other therapy efficacy

Except the three therapies mentioned above, the application of gut microbiota may also enhance the efficacy of other therapies in HCC. For instance, a temperature-responsive bacterial vector has been engineered that is capable of on-demand release of interleukin-15 (IL-15) complexed with its soluble receptor α (IL-15Rα) following microwave ablation (MWA) therapy. This biohybrid system demonstrated selective tumor tropism, undergoing thermal activation specifically within MWA-treated regions to achieve sustained local production of IL-15/IL-15Rα complexes. By combining MWA with engineered bacteria, tumor growth can be effectively inhibited and animal survival can be prolonged even in cases of incomplete MWA[156].

## Therapeutic interventions targeting gut microbiota for hepatocellular carcinoma

### Probiotics

**Figure 8.**
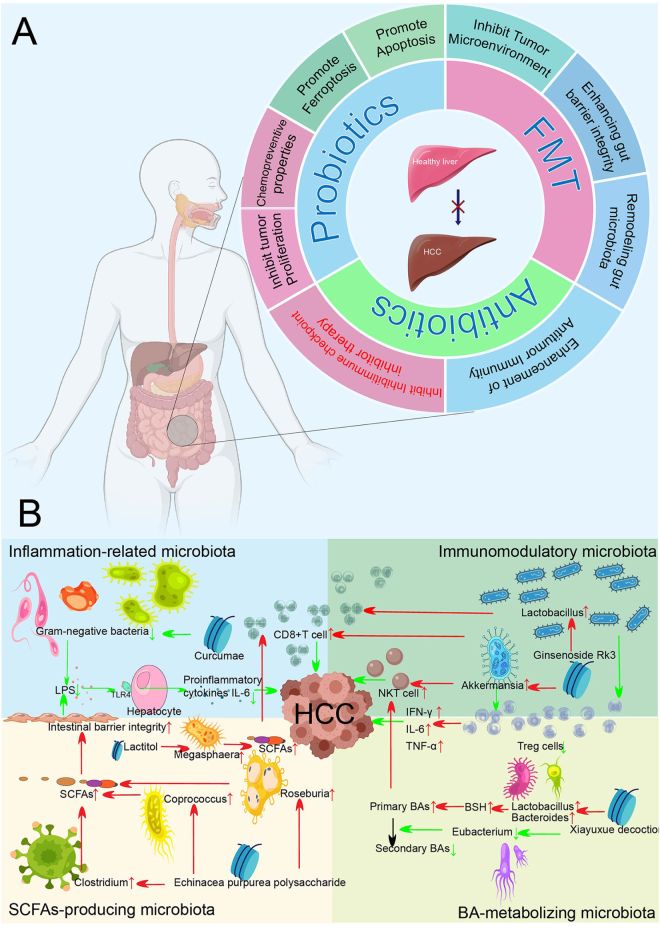
(A) Molecular mechanisms of targeting gut microbiota to intervene in hepatocellular carcinoma; (B) The mechanisms by which small molecules or traditional Chinese medicine inhibit HCC via gut microbiota.

Probiotics exhibit significant potential in the treatment and prevention of HCC through multitarget regulatory mechanisms (Fig. 8). Studies have demonstrated that probiotics can directly induce apoptosis of hepatoma cells by intervening in key signaling pathways. For instance, *Bacillus coagulans* MZY531 and *Bacillus subtilis* Z15 activate the mitochondrial apoptotic pathway by inhibiting the phosphorylation of the PI3K/AKT/mTOR pathway and upregulating the Bax/Bcl-2 ratio and caspase-3 activity, while bioactive peptides produced by *Bacillus velezensis* PM35 further enhance the apoptotic effect^[157–159]^. At the metabolic level, the probiotic cocktail Prohep enhances SCFAs production by stimulating propionic acid metabolism, whereas combining *Bifidobacterium bifidum* and *Lactobacillus plantarum* with *Salviae miltiorrhizae* polysaccharides elevates acetate and butyrate concentrations, improving insulin sensitivity^[160,161]^. *Bifidobacterium pseudolongum* metabolites, particularly acetate, activate hepatocyte GPR43 receptors, suppressing the IL-6/JAK1/STAT3 signaling cascade^[160–162]^. *Lactobacillus acidophilus* secretes valeric acid, which binds GPR41/43 to block the Rho-GTPase pathway and attenuate MASLD-HCC progression[163]. In immune modulation, *E. faecium* stimulates CD8+ T cells to produce interferon-γ via extracellular polysaccharides, synergizing with the ferroptosis inducer sorafenib to suppress SLC7A11 expression and amplify lipid peroxidation sensitivity[147]. *Lactobacillus reuteri* FLRE5K1 enhances antitumor immunity by driving Th1 cell polarization[164]. *Akkermansia muciniphila* induces hepatic CXCR6 + NKT cell accumulation while diminishing macrophage infiltration[125]. Targeting oncogenic signaling pathways, *Lactobacillus rhamnosus* and *B. pseudolongum* inhibit IL-6/STAT3 pathway activity by reducing IL-6 levels or enhancing STAT3 methylation, respectively, and thus blocking tumor cell growth and angiogenesis^[162,165]^. In the field of chemical toxin prevention, the combination of *L. rhamnosus* LC705 and *Propionibacterium freudenreichii* reduces aflatoxin bioavailability, while *L. rhamnosus* GG and *Lactobacillus casei* Shirota inhibit TP53 mutation-driven HCC development through adsorption and downregulation of oncogenic gene expression^[166,167]^. These findings systematically untangle the multifaceted mechanisms of probiotics in intervening in HCC progression, including apoptosis induction, metabolic remodeling, immune activation, and oncogenic signal inhibition. Simultaneously, they highlight the unique value of probiotics in enhancing the sensitivity of traditional therapies and early prevention, laying a theoretical foundation for the development of comprehensive treatment strategies for HCC based on precise microbiota regulation.

### Antibiotics

Antibiotic treatment profoundly disrupts gut microbiota composition, triggering dysbiosis that significantly influences host immune responses and tumor progression through bidirectional regulatory mechanisms. In HCC models, broad-spectrum antibiotic combinations (vancomycin, cefoperazone, ampicillin, neomycin, and metronidazole) induce colonic tuft cells to overexpress IL-25, which promotes macrophage alternative activation and CXCL10 secretion within the TME, ultimately accelerating HCC progression[79]. This dysbiosis also diminishes chemotherapy efficacy; antibiotic-mediated depletion of commensal *Lachnospiraceae* reduces treatment sensitivity by impairing colonization resistance, antitumor immunity, and drug metabolism[168]. Immunotherapy effectiveness similarly suffers from antibiotic-induced dysbiosis across tumor models, particularly through compromised symbiotic bacterial signals necessary for T cell priming and TME infiltration. For CTLA-4 blockade therapy, reintroducing *Bacteroides fragilis* or transferring *B. fragilis*-specific T cells restores therapeutic efficacy[169]. Furthermore, antibiotics substantially influence the therapeutic outcomes of ICIs[170]. A multicenter clinical trial of 4098 HCC patients receiving molecular targeted therapy or ICIs showed that antibiotic exposure correlated with reduced median PFS and OS, particularly in the ICI-treated group[171]. In a separate study of 395 advanced HCC patients, concurrent antibiotic use within 30 days of initial ICIs administration significantly increased cancer-related mortality, with stronger effects observed for antibiotics targeting aerobic bacteria[172]. Immunotherapy responders typically exhibit gut microbiota enriched in *Akkermansia, Ruminococcus*, and *Bifidobacterium* species^[137,173,174]^. Broad-spectrum antibiotics disrupt this balance by favoring *Bacteroides* and commensal Clostridia proliferation[175]. These shifts subsequently enhance the recruitment and activation of immunosuppressive cells – especially Tregs and MDSCs – which mechanistically contribute to diminished ICIs efficacy[176]. However, some targeted antibiotics demonstrate protective effects. Vancomycin monotherapy selectively eliminates Clostridium cluster XIVa, inhibiting bacterial 7α-dehydroxylation and accumulating PBAs. These PBAs enhance CXCL16 expression in liver sinusoidal endothelial cells, recruiting CXCR6^+^ NKT cells whose IFN-γ production suppresses HCC progression^[97,177,178]^. This protection stems from microbial community restructuring, decreased harmful metabolites like DCA, and PBA-driven NKT cell activation. Ultimately, antibiotic modulation exerts context-dependent immunological and oncological outcomes dictated by the specific dysbiosis pattern, underscoring the intricate crosstalk between microbiota and immunity. These insights support the development of precision antimicrobial interventions targeting discrete microbial populations.

### Fecal microbiota transplantation

FMT exhibits potential therapeutic value in inhibiting the progression of HCC through multidimensional modulation of gut microbiota homeostasis and the host’s immune-metabolic network (Fig. 8). Studies have demonstrated that FMT can suppress liver inflammation by regulating the intestinal immune microenvironment. For instance, it enhances the expression of CXCR6 in Group 3 innate lymphoid cells and promotes their liver homing mediated by the CXCL16/CXCR6 axis, leading to increased IL-22 secretion and reduced hepatic steatosis[179]. Simultaneously, FMT modulates the proportion of T-cell subsets, decreasing Th17 cells and IL-17A levels, thereby inhibiting the release of pro-inflammatory factors and angiogenic mediators, and disrupting the formation of the HCC microenvironment[11]. In terms of intestinal barrier repair, FMT enhances the expression of tight junction protein ZO-1, improves intestinal mucosal integrity, and reduces intestinal permeability[180]. This leads to decreased bacterial translocation and metabolite influx into the liver, ultimately mitigating the malignant transformation of MASLD into HCC[181]. Furthermore, regarding microbiota remodeling, FMT significantly increases the abundance of beneficial bacteria such as *Faecalibacterium, Bifidobacterium, Lactobacillus*, Christensenellaceae, Prevotellaceae, and Clostridiaceae[180]. It also suppresses the proliferation of pro-inflammatory bacteria like *Neisseria*, Odoribacter, and Oscillibacter[182]. In patients with liver cirrhosis, FMT increases the abundance of Burkholderiaceae and decreases that of Acidaminococcaceae, restoring a microbiota steady state dominated by Firmicutes and Actinobacteria^[183,184]^. Additionally, FMT reverses antibiotic resistance in patients with liver cirrhosis by reducing the abundance of vancomycin and beta-lactam resistance genes, thereby enhancing the potential of antimicrobial therapy^[185,186]^. Although direct research on HCC is currently limited, FMT may indirectly inhibit the development and progression of HCC through mechanisms such as regulating the IL-22/IL-17 balance, repairing the intestinal barrier, and reversing microbiota dysbiosis. The mechanisms of FMT are closely related to the functional remodeling of the gut microbiota-liver axis, and further exploration is needed to understand its potential application in the regulation of the HCC immune microenvironment and combination therapies.

### Targeting specific microbiota with small molecules or traditional Chinese medicine

The pivotal role of microbial communities in modulating HCC pathogenesis has accelerated the advancement of advanced therapeutics targeting specific microbiota via small molecules or traditional Chinese medicine (TCM), demonstrating efficacy in animal or human studies (Fig. 8 and Supplementary Digital Content Figure S6, available at: http://links.lww.com/JS9/F152). According to the mechanisms involved in the development of HCC, the targeted microbial populations primarily encompass immunomodulatory, SCFAs-producing, BA-metabolizing, inflammation-related microbiota. Particular bacteria may be involved in more than one mechanism mentioned above (Supplementary Digital Content Table S2, available at: http://links.lww.com/JS9/F153).

Among immunomodulatory microbiota, Akkermansia species critically influence antitumor immune responses by promoting the number and activity of NKT cells, increasing the proportion of CD8+ T cells, and decreasing the infiltration of immunosuppressive cells[187]. *Akkermansia muciniphila* have also been reported that it can increase the levels of IFN-γ, IL-6, TNF-α via decreasing the proportion of CD4 + CD25 + Foxp3 + Treg in the peripheral blood and spleen, and thus enhancing the antitumor effect of cisplatin in lung cancer mice model. Compounds such as astaxanthin[188], ginsenoside Rk3[189], Huaier polysaccharides[190], dextran-carbenoxolone conjugates (DEX-CBX)[108], and xierezhuyubuxu decoction[191], can upregulate Akkermansia abundance, thereby suppressing HCC. Beyond Akkermansia, stigmasterol can also enhance anti-HCC immunity (reducing Tregs/CD8^+^ T cells ratios in the intestine and tumor tissue) by increasing *Lactobacillus johnsonii, L. murinus*, and *L. reuteri* populations. Similarly, 2,5-dimethylcelecoxib downregulates PD-1 expression and upregulates IFN-γ production in NK and T cells via gastrointestinal microbiota modulation[192]. *Lactobacillus*, a well-known probiotics, can be enriched by curcumae[193], DEX-CBX[108], ganfule capsules[194], ginseng glucosyl oleanolate[195], ginsenoside Rk3[189], Jianpi–Huatan–Huoxue–Anshen formula[196], *Morus nigra L.* leaves derived nanoparticles[197], nimbolide[198], stigmasterol[192], tomato powder[199], ulva lactuca ulvan[200], and xiayuxue decoction[92]. Conversely, *Grifola frondosa* polysaccharide-protein complexes[201] reduce *Lactobacillus*. Notably, *L. brevis* alleviates HCC progression[202], while *L. acidophilus* potentiates oncolytic virotherapy[203]. Enriched *Lachnoclostridium* or *Lachnospiraceae*, and decreased Prevotella_9 correlate with better immunotherapy responses[139], which can be upregulated by Xiayuxue decoction (*Lachnospiraceae*, Prevotella_9)[204], Jiawei Xiaoyao San (*Lachnospiraceae*)[205], ginsenoside Rk3 (*Lachnospiraceae*)[189], DEX-CBX (*Lachnospiraceae*)[108], and berberine (*Lachno clostridium, Lachnospiraceae*)[206]. Lactitol can downregulate *Lachnoclostridium*[207].

SCFAs-producing microbiota (*Coprococcus, Clostridium*, and *Roseburia*) are enhanced by *Echinacea purpurea* polysaccharide[208], which reinforces intestinal barrier integrity via tight junction proteins, reduces LPS leakage, and suppresses TLR4/NF-κB signaling. This cascade inhibits pro-inflammatory cytokines (e.g., IL-6) and migration factors (e.g., MMP-2), ultimately curtailing HCC. Similar up-regulated effects for some of those microbiota also occur with berberine (*Roseburia*)[206], Brassica rapa polysaccharides (*Clostridium*)[209], ginseng glucosyl oleanolate (*Clostridium*)[195], Jianpi–Huatan–Huoxue–Anshen formula (*Clostridium*)[196], *Panax ginseng* (*Coprococcus* and *Roseburia*)[210], Shaoyao Ruangan Mixture (*Clostridium*)[211]. Butyrate producers *Turicibacter* and *Roseburia* can also be upregulated by oral exosome-like nanovesicles from *Phellinus linteus* (*Turicibacter*)[212], *M. nigra L.* leaves derived nanoparticles (*Turicibacter*)[197], Shaoyao Ruangan Mixture (*Turicibacter*)[211], Berberine (*Roseburia*)[206], *Echinacea purpurea* polysaccharide (*Roseburia*)[208], and *P. ginseng* (*Roseburia*)[210]. Megasphaera, which can be upregulated by Lactitol[207], can regulate the response of CD8-positive T cells and enhance the effects of cancer immunotherapy by inducing SCFAs such as butyrate and pentanoate. Muribaculaceae can also generate the SCFAs, which can be upregulated by berberine[206], grifola frondosa polysaccharide-protein complexes[201], THSWD (Tao Hong Si Wu Decoction)[213], selenium-rich royal jelly[214], and can be down-regulated by oral exosome-like nanovesicles from Phellinus linteus[212]. Human microbiota, such as *Erysipelotrichaceae* and *Lachnospiraceae*, can enhance antitumor immunity by generating formate[215], which can be enhanced by astaxanthin (Faecalibaculum)[188], Huaier polysaccharides (Ileibacterium and Allobaculum)[190], and decreased by safflower yellow[216], Huaier polysaccharides (Dubosiella)[190], and ulva lactuca ulvan (Holdemania)[200].

In BA metabolism, Xiayuxue decoction elevates BSH activity via increasing *Bacteroides* and *Lactobacillus* abundance[92]. BSH generates PBAs, while reduced *Eubacterium* limits their conversion to SBAs. Accumulated PBAs activate hepatic NKT cells to secrete IFN-γ,[92]conferring anti-HCC immunity. 2,5-Dimethylcelecoxib also upregulates IFN-γ in NK/T cells by enriching *Bacteroides acidifaciens, Odoribacter laneus*, and *O. splanchnicus*[217]. Odoribacter is also increased by THSWD[213]. *Bacteroides*, the dominant beneficial bacteria that inhibit pathogenic bacteria, can also be upregulated by berberine[206], ginseng glucosyl oleanolate[195], ginsenoside Rk3[189], Huaier polysaccharides[190], *Pholiota adiposa*[218], recombinant phycoerythrin[219], tomato powder[199], and Xiayuxue decoction[92], but downregulated by celastrol (*B. fragilis*)[220], curcumae[193], Ganfule capsules[194], Jiawei Xiaoyao San[205], nimbolide[198], *P. ginseng*[210], safflower yellow[216], Shaoyao Ruangan Mixture[211], and Xiayuxue decoction[204]. Firmicutes, another dominant beneficial bacteria, can be increased with the application of Ganfule capsules[194], Jiawei Xiaoyao San[205], stigmasterol[192], *M. nigra L.* leaves derived nanoparticles[197], and tomato powder[199], but be decreased by ginseng glucosyl oleanolate[195], ginsenoside Rk3[189], recombinant phycoerythrin[219], and Xiayuxue decoction[92]. The roles of *Bacteroides* and Firmicutes in HCC warrant further investigations.

Pertaining to inflammation-related microbiota, Xiayuxue decoction suppresses Enterobacteriaceae[204], and thus amelioratin pro-tumorigenic inflammation and hepatocarcinogenesis[221]. Streptococcus is another inflammation-related microbiota and may be involved in the development of HCC[222], which can be upregulated by *P. ginseng*[210]. THSWD enriches Duncaniella, whose metabolite glabrol induces lysosomal autophagy-mediated apoptosis in HCC cells[213]. Anti-inflammatory *Prevotella* and Oscillibacter inhibit HCC[223] and can be upregulated by berberine (Oscillibacter)[206], ginsenoside Rk3 (Oscillibacter)[189], and Xiayuxue decoction (*Prevotella, Prevotellaceae*, Prevotella_9, and Prevotellaceae_NK3B31_group)[92], but downregulated by Jiawei Xiaoyao San (Oscillibacter)[205], *P. adiposa* (Prevotellaceae_UCG−001 and UCG−003)[218], and recombinant phycoerythrin (*Prevotella*)[219]. *B. longum*-derived extracellular vesicles have been reported that it can prevent HCC by alleviating liver fibrosis, apoptosis, oxidative stress and modulating the TGF-β1/Smad signaling[224]. *Bifidobacterium* can be up-regulated by Curcumae, Ginsenoside Rk3[189], Lactitol, Nimbolide, and Tomato Powder. Curcumae treatment can reduce gram-negative bacteria tendency and suppresse LPS production. LPS can promote HCC by inducing the secretion of pro-inflammatory cytokines like IL-6. Therefore, the inhibition of LPS production by curcumae will inhibit the development of HCC. The pro-inflammatory *Helicobacter*, which can disrupt immunity and microbiota balance[218], is reduced by DEX-CBX[108] and ginsenoside Rk3[189]. In summary, numerous small molecules and TCM have been indicated that they may hold the ability to inhibit HCC via modulating particular microbiota. The different species of microbiota in the same family or genus may hold different roles for HCC development, and thus further research is warranted to elucidate the precise mechanisms of these particular microbiota in HCC progression and optimize particular microbiota-targeted therapeutic applications.

## Navigating limitations and individualized challenges in microbiota-targeted therapies for hepatocellular carcinoma

The outlook for microbiota-targeted therapies in HCC is optimistic but several individualized challenges must be overcome before widespread clinical implementation can be realized.

To evaluate the role of microbiota in HCC, a standardized approach for testing and reporting is significant. Lack of standardized protocols for sample selection, collection, processing, sequencing platforms, and bioinformatic analysis technologies contributes to inconsistent and potentially biased outcomes. For instance, though microbiota derived from the colonic mucosa and feces share similarities, critical differences exist[225]; relying solely on one sample type may obscure true microbial associations with cancer. Moreover, incorrect collection or processing of samples may render microbiota susceptible to contamination, especially for low-biomass microbiota[226]. Thus, multisite sampling and stringent anticontamination methods (e.g., sterile protocols) are essential for rigorous and exact outcomes. Concurrently, the establishment of comprehensive, consensus-driven standardized operating procedures across the researches is urgently warranted to enhance data homogeneity and cross-study comparability, which may reduce the generation of contradictory results within basic research. Furthermore, interpreting not association but causality remains difficult. While rich correlative data link microbial profiles to HCC risk or progression (frequently generated via sequencing and bioinformatics), definitive causality of a particular bacterium for HCC development requires further researches, like FMT or single strain bacteria transplantation. As mentioned above, we should focus on the level of bacterial strain to clarify the functions of the microbial community.

Beyond methodology, intrinsic biological variation in microbiota contributes to profound individualized challenges. Individualized differences in microbiota composition and function are influenced by numerous factors, like host genetics, dietary patterns, age, sex, geographical variation, and comorbid diseases^[227,228]^. The impact of geographical variation for microbiota is particularly striking and multifaceted, often reflecting underlying disparities in socioeconomic status, dietary patterns, and local environments; one significant beneficial microbiota in one population may not also be significantly beneficial for other populations in another region with distinct microbial ecology, limiting the generalizability of microbiota-targeted therapies in HCC. As we mentioned above, the different etiology-associated subtypes of HCC may hold significantly different microbiota compositions, and the individualized differences of microbiota composition and function in different subtypes of HCC may warrant further validation using larger cohorts. Moreover, basic research often relies on animal models (such as sterile mice and chemically induced models), which can easily control variables and reveal mechanisms, but it is difficult to fully reflect the complex human intestinal environment and host background, partly explaining the existing significant discrepancies between basic and clinical studies. For instance, animal models indeed demonstrate that DCA promotes hepatocarcinogenesis in high-fatdiet-induced HCC through reactive oxygen species-mediated DNA damage[45]. Still, it has been found that conjugated DCA, glyco-DCA, can remarkably inhibit the growth of HCC *in vivo* and *in vitro*[46]. Antibiotics can inhibit HCC in animal studies, but antibiotics are recognized as a significant contributor to ICI resistance in clinical studies. Emerging sophisticated models and technologies, such as patient-derived organoids, patient-derived xenograft, and single-microbe sequencing (analogous to single-cell sequencing), offer promising avenues to dissect individual strain-level contributions and validate mechanisms *in vitro* within a personalized context.

The translation from basic science to clinical practice also encounters other significant barriers. The highly personalized nature of host–microbe interactions results in variable therapeutic sensitivity across patients to the same microbial agents. We can further enhance the potential of microbiota-targeted therapy in HCC by combining advanced materials or engineering specific bacteria.

## Conclusion

Over the past three decades, research has systematically explored the role of gut microbiota in the development of HCC, revealing a multidimensional regulatory network and highlighting its significant therapeutic potential. Current evidence indicates that imbalances in gut microbiota can promote the progression of HCC especially via the metabolism–immunity axis. For instance, carcinogenic metabolites, such as DCA and TMA, produced by dysbiotic microbiota enter the liver through the enterohepatic circulation, leading to metabolic reprogramming of liver cells and remodeling of the tumor immune microenvironment. However, significant gaps persist in our understanding of the gut microbiota’s key regulatory mechanisms on liver glucose and amino acid metabolism pathways, particularly how microbiota-derived signals influence metabolic enzyme activity through epigenetic modifications or organelle function regulation. Moreover, precision intervention strategies targeting the particular gut bacterium are explored to enhance efficacy in immunotherapy, molecular targeted therapy, conventional chemotherapy, and surgery for HCC. Until now, the promising microbiota-targeted therapies include oral probiotics, antibiotics, FMT, particular small molecules, and TCM. These advancements represent a novel approach for individualized HCC treatment. However, current microbiota-targeted therapies still encounter clinical translation challenges, including the need for standardized donor screening in microbiota transplantation, understanding recipient heterogeneity response mechanisms, and addressing the risk of potential pathogenic bacterial translocation[229]. More clinical trials are urgently needed to evaluate these issues and facilitate the successful transition of microbiota-targeted therapies from the research setting to clinical practice.

## Supplementary Material

## Data Availability

All data generated and analyzed during this study are included in this article. The data supporting the findings of this study are available from the corresponding author upon reasonable request.

## Research registration unique identifying number (UIN)

No registration needed.
