# Supplementary figures and images for: The landscape of gut microbiota in hepatocarcinogenesis: a comprehensive review of pathogenesis and therapeutic interventions
Source: Int J Surg. 2025 Sep 22;112(1):1673–95. doi: 10.1097/JS9.0000000000003511 (PMC12825765; doi:10.1097/JS9.0000000000003511)

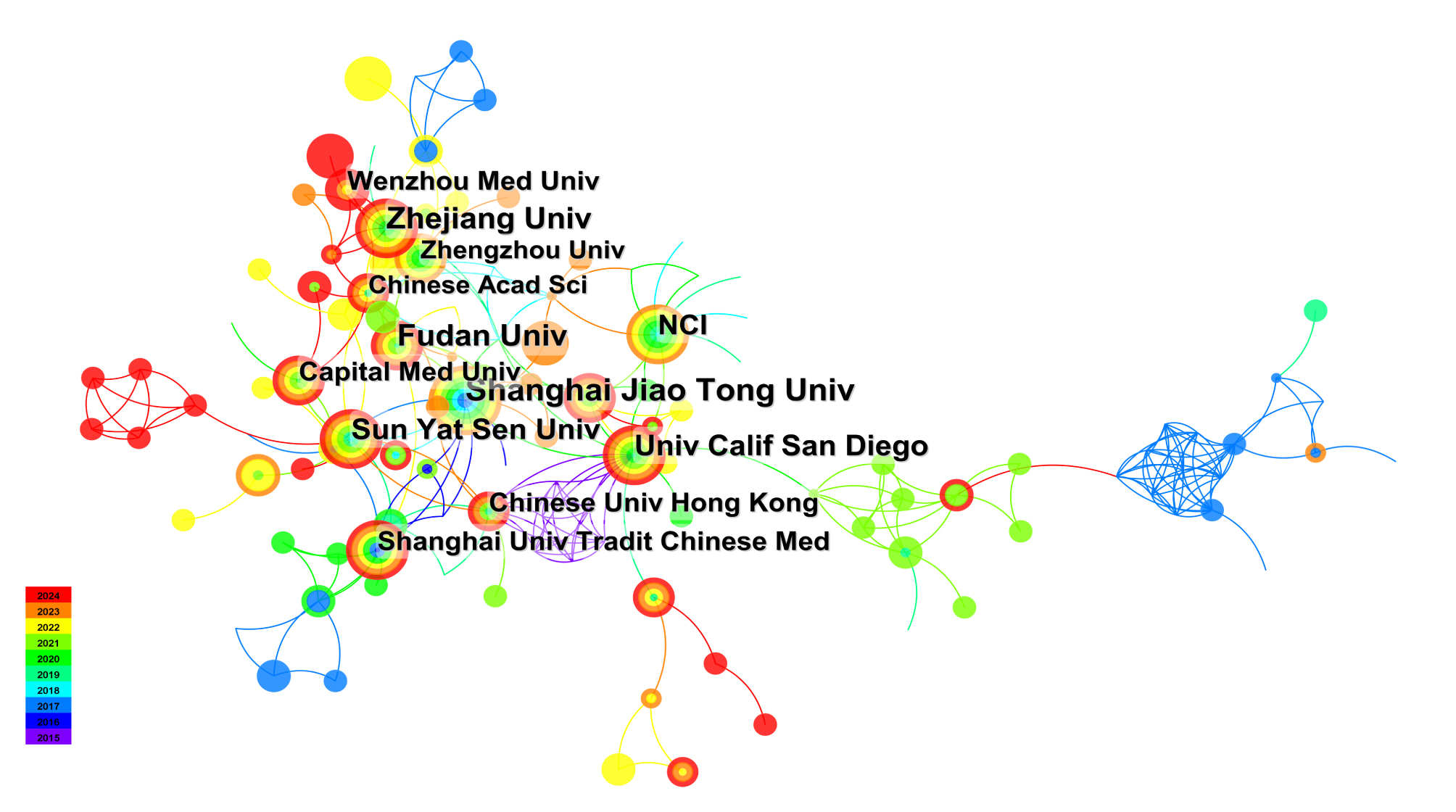
Figure S1


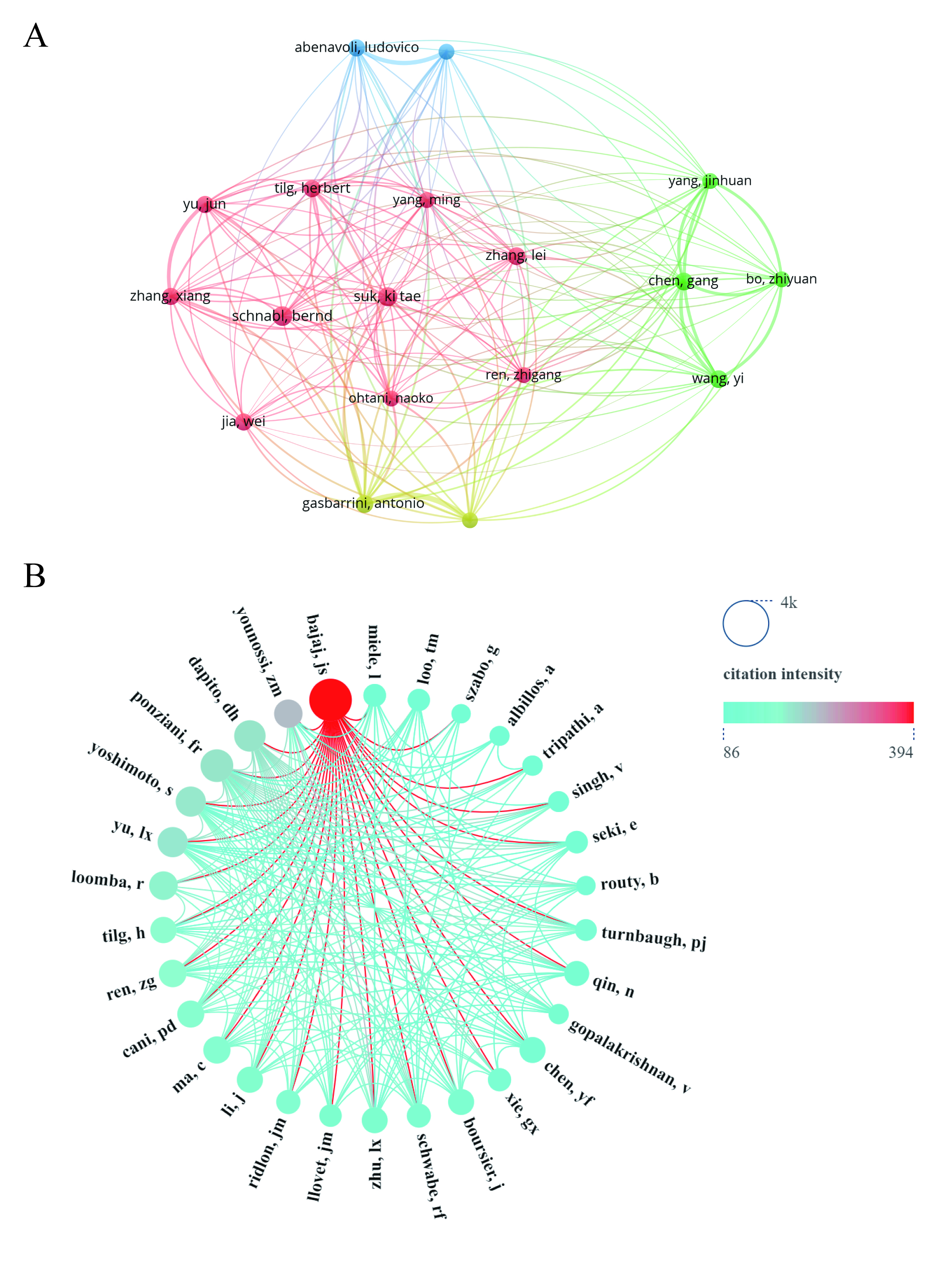
Figure S2


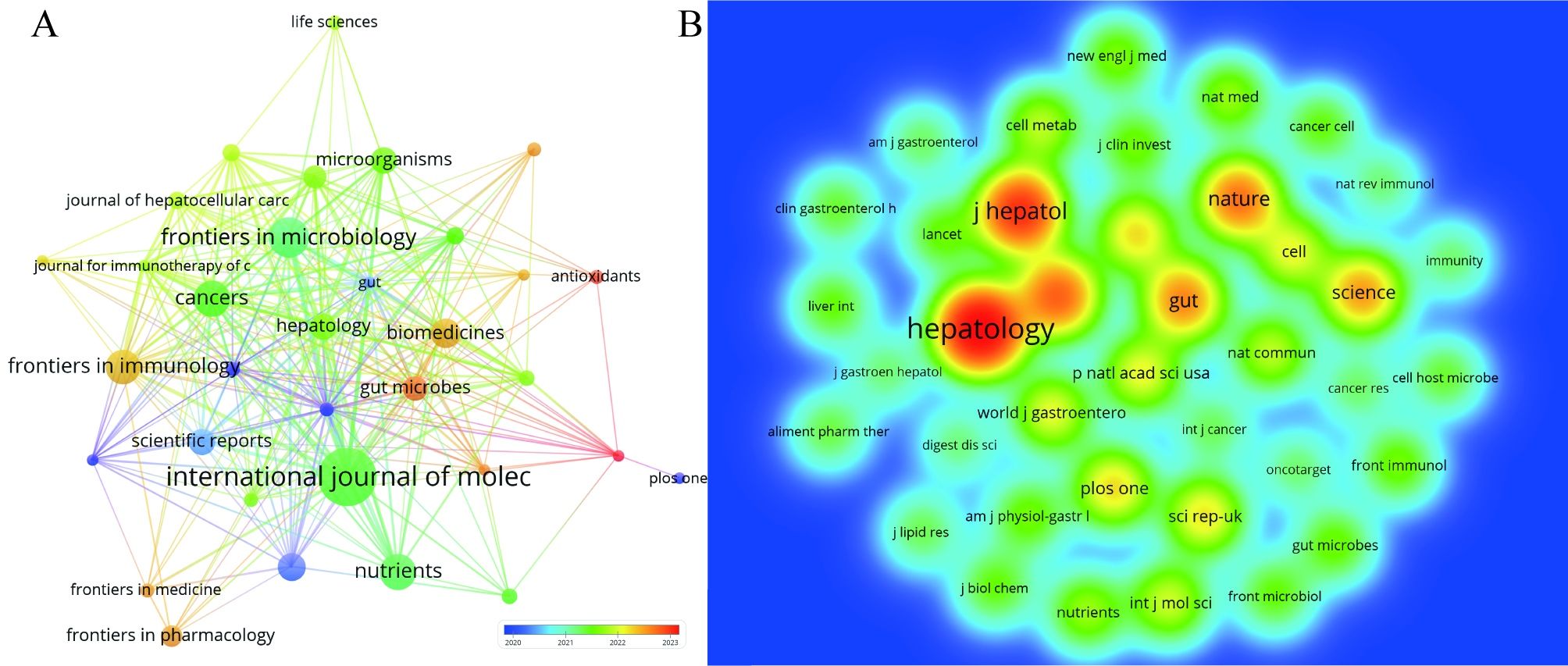
Figure S3


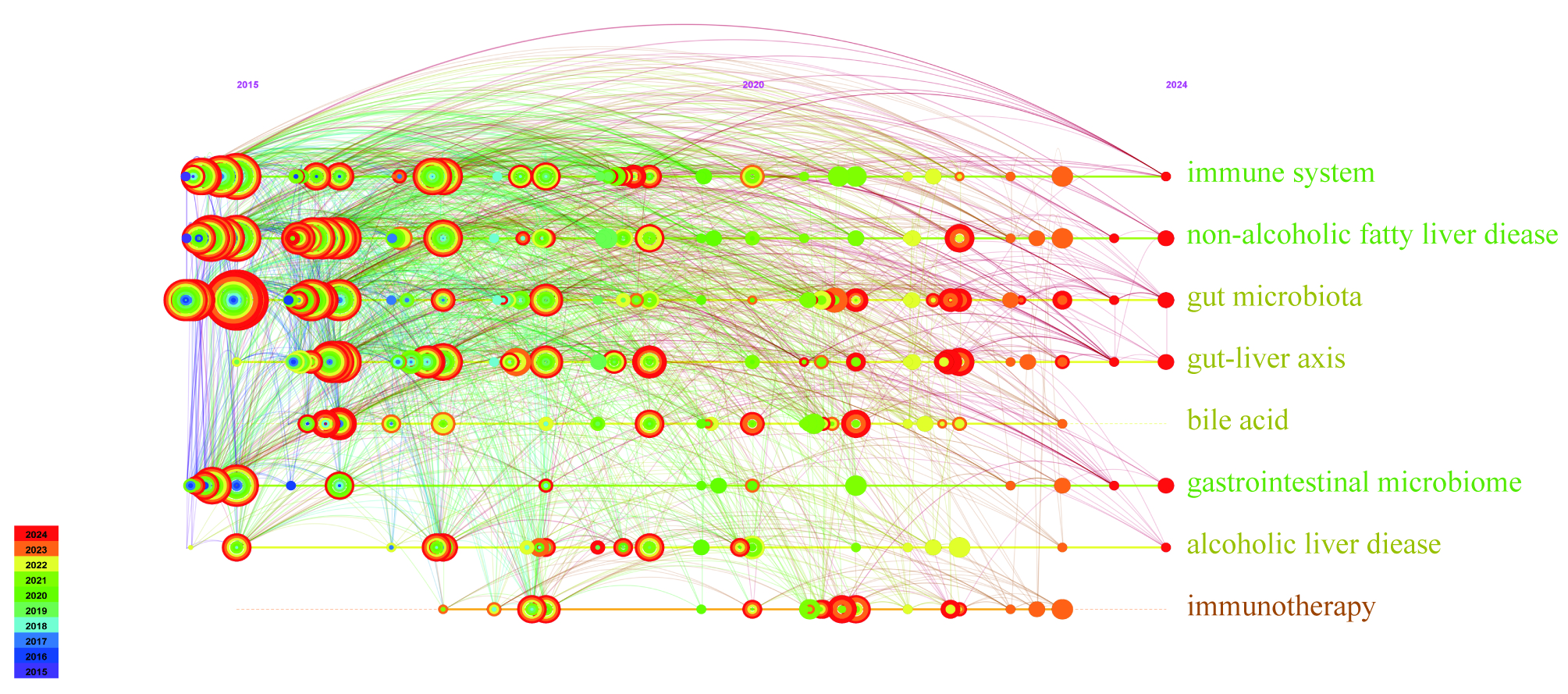
Figure S4


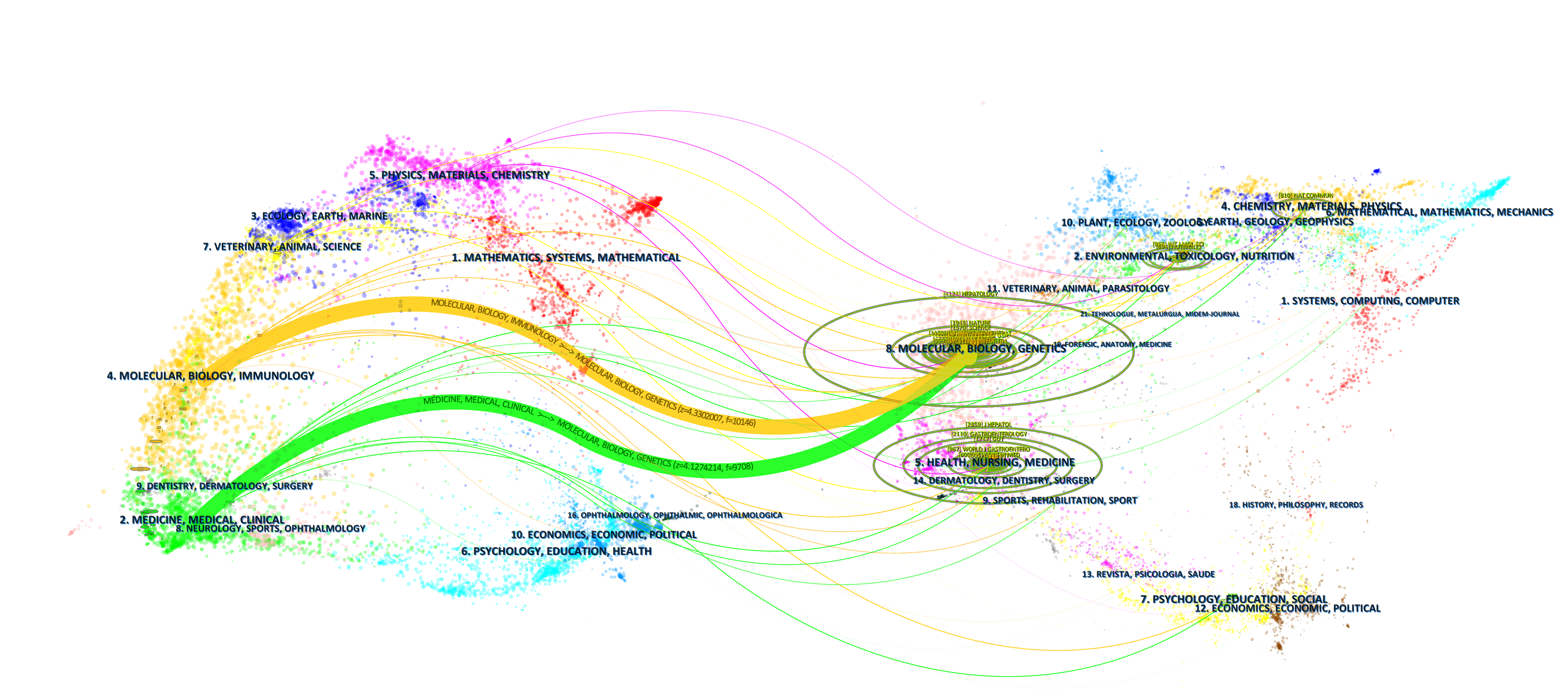
Figure S5


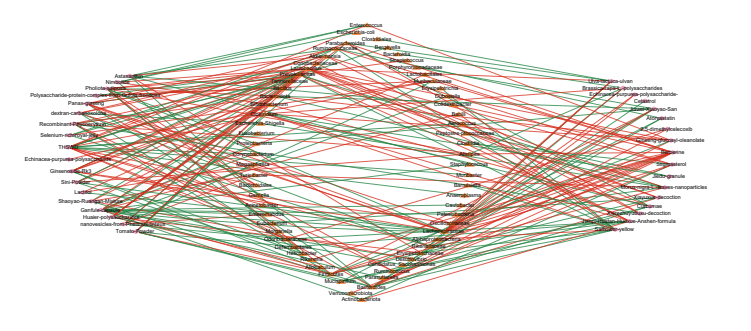
Figure S6

Supplement: Supplementary file 1 [file js9-112-1673-001.docx]
