# Supplementary material for: The landscape of gut microbiota in hepatocarcinogenesis: a comprehensive review of pathogenesis and therapeutic interventions
Source: Int J Surg. 2025 Sep 22;112(1):1673–95. doi: 10.1097/JS9.0000000000003511 (PMC12825765; doi:10.1097/JS9.0000000000003511)
Supplement: Supplementary file 2 [file js9-112-1673-002.docx]

**Supplementary Table 1. Gut Microbiota-derived Metabolites (and Metabolizing Enzymes)**

|  | Phylum | Class | Order | | Family | Genus | Species / Strain |  | |
| --- | --- | --- | --- | --- | --- | --- | --- | --- | --- |
| BSH |  |  |  |  | |  |  |  |  |
| BSH | Actinomycetota | Actinobacteria | Bifidobacteriales | | Bifidobacteriaceae | *Bifidobacterium* | *pseudocatenulatum* | [1] | |
| BSH | Actinomycetota | Actinobacteria | Bifidobacteriales | | Bifidobacteriaceae | *Bifidobacterium* | *longum* | [2] | |
| BSH | Actinomycetota | Actinobacteria | Bifidobacteriales | | Bifidobacteriaceae | *Bifidobacterium* | *breve* | [3] | |
| BSH | Ascomycota | Saccharomycetes | Saccharomycetales | | Saccharomycetaceae | *Saccharomyces* | *boulardii* (strain) | [4] | |
| BSH | Bacteroidota | Bacteroidia | Bacteroidales | | Bacteroidaceae | *Bacteroides* | *dorei* (BDX-01 strain) | [5] | |
| BSH | Bacteroidota | Bacteroidia | Bacteroidales | | Bacteroidaceae | *Bacteroides* | *fragilis* | [2, 6, 7] | |
| BSH | Bacteroidota | Bacteroidia | Bacteroidales | | Bacteroidaceae | *Bacteroides* | *ovatus* | [6] | |
| BSH | Bacteroidota | Bacteroidia | Bacteroidales | | Bacteroidaceae | *Bacteroides* | *thetaiotaomicron* | [6] | |
| BSH | Bacteroidota | Bacteroidia | Bacteroidales | | Bacteroidaceae | *Bacteroides* | *uniformis* | [6] | |
| BSH | Bacteroidota | Bacteroidia | Bacteroidales | | Bacteroidaceae | *Bacteroides* | *vulgatus* | [6] | |
| BSH | Bacillota | Bacilli | Bacillales | | Bacillaceae | *Bacillus* | *cereus* | [8] | |
| BSH | Bacillota | Bacilli | Lactobacillales | | Lactobacillaceae | *Lacticaseibacillus* | *casei* | [9] | |
| BSH | Bacillota | Bacilli | Lactobacillales | | Lactobacillaceae | *Lacticaseibacillus* | *paracasei* | [3] | |
| BSH | Bacillota | Bacilli | Lactobacillales | | Lactobacillaceae | *Lacticaseibacillus* | *paracasei* | [9] | |
| BSH | Bacillota | Bacilli | Lactobacillales | | Lactobacillaceae | *Lacticaseibacillus* | *rhamnosus* (GG strain) | [10] | |
| BSH | Bacillota | Bacilli | Lactobacillales | | Lactobacillaceae | *Lacticaseibacillus* | *rhamnosus* | [9] | |
| BSH | Bacillota | Bacilli | Lactobacillales | | Lactobacillaceae | *Lactiplantibacillus* | *plantarum* | [9] | |
| BSH | Bacillota | Bacilli | Lactobacillales | | Lactobacillaceae | *Lactobacillus* | (Genus level) | [11] | |
| BSH | Bacillota | Bacilli | Lactobacillales | | Lactobacillaceae | *Lactobacillus* | *gasseri* (RW2014 strain) | [12] | |
| BSH | Bacillota | Bacilli | Lactobacillales | | Lactobacillaceae | *Lactobacillus* | *gasseri* | [9] | |
| BSH | Bacillota | Bacilli | Lactobacillales | | Lactobacillaceae | *Lactobacillus* | *johnsonii* | [13] | |
| BSH | Bacillota | Bacilli | Lactobacillales | | Lactobacillaceae | *Lactobacillus* | *acidophilus* | [9] | |
| BSH | Bacillota | Bacilli | Lactobacillales | | Lactobacillaceae | *Lactobacillus* | *amylovorus* | [9] | |
| BSH | Bacillota | Bacilli | Lactobacillales | | Lactobacillaceae | *Lactobacillus* | *delbrueckii* | [9] | |
| BSH | Bacillota | Bacilli | Lactobacillales | | Lactobacillaceae | *Latilactobacillus* | *sakei* | [9] | |
| BSH | Bacillota | Bacilli | Lactobacillales | | Lactobacillaceae | *Ligilactobacillus* | *animalis* | [9] | |
| BSH | Bacillota | Bacilli | Lactobacillales | | Lactobacillaceae | *Ligilactobacillus* | *murinus* | [9] | |
| BSH | Bacillota | Bacilli | Lactobacillales | | Lactobacillaceae | *Ligilactobacillus* | *ruminis* | [9] | |
| BSH | Bacillota | Bacilli | Lactobacillales | | Lactobacillaceae | *Ligilactobacillus* | *salivarius* | [9] | |
| BSH | Bacillota | Bacilli | Lactobacillales | | Lactobacillaceae | *Limosilactobacillus* | *fermentum* | [9] | |
| BSH | Bacillota | Bacilli | Lactobacillales | | Lactobacillaceae | *Limosilactobacillus* | *mucosae* | [9] | |
| BSH | Bacillota | Bacilli | Lactobacillales | | Lactobacillaceae | *Limosilactobacillus* | *reuteri* | [4, 9] | |
| BSH | Bacillota | Bacilli | Lactobacillales | | Lactobacillaceae | *Limosilactobacillus* | *reuteri* | [4] | |
| BSH | Bacillota | Bacilli | Lactobacillales | | Lactobacillaceae | *Limosilactobacillus* | *reuteri* (NCIMB 30242) | [14] | |
| BSH | Bacillota | Erysipelotrichia | Erysipelotrichales | | Erysipelotrichaceae | *Turicibacter* | (Species unspecified) | [15] | |
| BSH | Verrucomicrobiota | Verrucomicrobiae | Verrucomicrobiales | | Verrucomicrobiaceae | *Akkermansia* | *muciniphila* | [16] | |
| 7α-dehydroxylating |  |  |  | |  |  |  |  | |
| 7α-dehydroxylating | Bacillota | Clostridia | Clostridiales | | Lachnospiraceae | *Clostridium* | *scindens* | [17] | |
| 7α-dehydroxylating | Bacteroidota | Bacteroidia | Bacteroidales | | Muribaculaceae | *Claveliimonas* | *bilis* (new genus) | [18] | |
| 7α-dehydroxylating | Bacillota | Eubacteria | Eubacteriales | | Eubacteriaceae | *Eubacterium* | sp. c-25 | [19] | |
| 7α-dehydroxylating | Bacillota | Clostridia | Clostridiales | | Lachnospiraceae | *Clostridium* | *hiranonis* | [20] | |
| 7α-dehydroxylating | Bacillota | Clostridia | Clostridiales | | Lachnospiraceae | *Clostridium* | sp. TO-931 | [21] | |
| TMA |  |  |  | |  |  |  |  | |
| TMA | Bacteroidota | Bacteroidia | Bacteroidales | | Prevotellaceae | *Prevotella* | *copri* | [22] | |
| TMA | Bacillota | Clostridia | Clostridiales | | Clostridiaceae | *Clostridium* | *difficile* | [23] | |
| TMA | Bacillota | Clostridia | Clostridiales | | Ruminococcaceae | *Emergencia* | *timonensis* | [24] | |
| TMA | Bacillota | Clostridia | Clostridiales | | Ruminococcaceae | *Ihubacter* | *massiliensis* | [24] | |
| TMA | Bacillota | Clostridia | Clostridiales | | Ruminococcaceae | *Ruminococcus* | (Genus level) | [25] | |
| TMA | Bacillota | Clostridia | Clostridiales | | Ruminococcaceae | (Family level) |  | [26] | |
| TMA | Bacillota | Clostridia | Clostridiales | | (Order level) |  |  | [25] | |
| TMA | Pseudomonadota | Gammaproteobacteria | Enterobacterales | | Enterobacteriaceae | *Citrobacter* | *amalonaticus* | [27] | |
| TMA | Pseudomonadota | Gammaproteobacteria | Enterobacterales | | Enterobacteriaceae | (Family level) |  | [28] | |
| TMA | Pseudomonadota | Gammaproteobacteria | Enterobacterales | | Enterobacteriaceae | *Escherichia* | (Genus level) | [29] | |
| TMA | Pseudomonadota | Gammaproteobacteria | Enterobacterales | | Enterobacteriaceae | *Klebsiella* | (Genus level) | [29] | |
| TMA | Pseudomonadota | Gammaproteobacteria | Enterobacterales | | Enterobacteriaceae | *Proteus* | (Genus level) | [23] | |
| TMA | Pseudomonadota | Gammaproteobacteria | Enterobacterales | | Enterobacteriaceae | *Shigella* | (Genus level) | [23] | |
| TMA | Pseudomonadota | Gammaproteobacteria | Pseudomonadales | | Moraxellaceae | *Acinetobacter* | *baumannii* | [30] | |
| SCFA |  |  |  | |  |  |  |  | |
| Propionate | Bacillota | Clostridia | Clostridiales | | Clostridiaceae | *Clostridium* | *bifermentans* (XIV ASF 356, 492) | [31] | |
| Propionate | Bacillota | Clostridia | Clostridiales | | Clostridiaceae | *Clostridium* | *ramosum* | [31] | |
| Acetate | Bacillota | Clostridia | Clostridiales | | Clostridiaceae | *Clostridium* | *bifermentans* (XIV ASF 356, 492) | [31] | |
| Acetate | Bacillota | Clostridia | Clostridiales | | Clostridiaceae | *Clostridium* | *ramosum* | [31] | |
| Acetate | Bacillota | Bacilli | Lactobacillales | | Lactobacillaceae | *Limosilactobacillus* | *reuteri* | [32] | |
| Acetate | Actinomycetota | Actinobacteria | Bifidobacteriales | | Bifidobacteriaceae | *Bifidobacterium* | *pseudolongum* | [33] | |
| Butyrate | Bacillota | Clostridia | Clostridiales | | Lachnospiraceae | *Roseburia* | *cecical* | [34] | |
| Butyrate | Bacillota | Clostridia | Clostridiales | | Lachnospiraceae | *Roseburia* | *intestinalis* | [34] | |
| Butyrate | Bacillota | Eubacteria | Eubacteriales | | Eubacteriaceae | *Eubacterium* | *rectal* | [34] | |
| Butyrate | Bacillota | Clostridia | Clostridiales | | Ruminococcaceae | *Faecalibacterium* | *prausnitzii* | [34] | |
| Butyrate | Bacillota | Clostridia | Clostridiales | | Erysipelotrichaceae | *Faecalibaculum* | (Genus level) | [35] | |
| Butyrate | Bacillota | Clostridia | Clostridiales | | Ruminococcaceae | *Ruminococcus* | *torques* | [35] | |
| Butyrate | Bacillota | Clostridia | Clostridiales | | Peptostreptococcaceae | *Romboutsia* | (Genus level) | [35] | |
| SCFA | Actinomycetota | Actinobacteria | Bifidobacteriales | | Bifidobacteriaceae | *Bifidobacterium* | *longum* | [36] | |
| SCFA | Bacillota | Clostridia | Clostridiales | | Lachnospiraceae | *Clostridium* | *symbiosum* | [36] | |
| SCFA | Bacillota | Clostridia | Clostridiales | | Ruminococcaceae | *Faecalibacterium* | *prausnitzii* | [36] | |
| SCFA | Bacillota | Bacilli | Lactobacillales | | Lactobacillaceae | *Limosilactobacillus* | *fermentum* | [36] | |
| SCFA | Bacillota | Clostridia | Clostridiales | | Lachnospiraceae | *Dorea* | *formicigenerans* (ATCC 27755) | [37] | |
| SCFA | Bacillota | Clostridia | Clostridiales | | Lachnospiraceae | *Anaerostipes* | *hadrus* | [37] | |
| SCFA | Bacillota | Clostridia | Clostridiales | | Lachnospiraceae | (Unnamed) | 5_1_63FAA_u_t | [37] | |
| valeric acid | Bacillota | Bacilli | Lactobacillales | | Lactobacillaceae | *Lactobacillus* | *acidophilus* | [38] | |
| valeric acid | Verrucomicrobiota | Verrucomicrobiae | Verrucomicrobiales | | Verrucomicrobiaceae | *Akkermansia* | *muciniphila* | [39] | |
| SCFAs | Bacillota | Bacilli | Lactobacillales | | Lactobacillaceae | *Lactiplantibacillus* | *plantarum* (J26 strain) | [40] | |
| SCFAs | Bacteroidota | Bacteroidia | Bacteroidales | | Muribaculaceae | (Family level) |  | [41] | |
| SCFAs | Bacteroidota | Bacteroidia | Bacteroidales | | Bacteroidaceae | *Bacteroides* | (Genus level) | [41] | |
| SCFAs | Desulfobacterota | Desulfovibrionia | Desulfovibrionales | | Deferribacteraceae | *Mucispirillum* | (Genus level) | [41] | |
| SCFAs | Bacillota | Bacilli | Lactobacillales | | Lactobacillaceae | *Lactobacillus* | (Genus level) | [41] | |
| Tryptophan |  |  |  | |  |  |  |  | |
| tryptophan | Actinomycetota | Actinobacteria | Bifidobacteriales | | Bifidobacteriaceae | *Bifidobacterium* | (Genus level) | [42] | |
| tryptophan | Actinomycetota | Actinobacteria | Bifidobacteriales | | Bifidobacteriaceae | *Bifidobacterium* | *bifidum* | [43] | |
| tryptophan | Bacillota | Bacilli | Lactobacillales | | Lactobacillaceae | *Lacticaseibacillus* | *rhamnosus* (HF01 strain) | [44] | |
| tryptophan | Bacillota | Bacilli | Lactobacillales | | Lactobacillaceae | *Lactobacillus* | *acidophilus* (KLDS1.0901 strain) | [45] | |
| tryptophan | Bacillota | Bacilli | Lactobacillales | | Lactobacillaceae | *Limosilactobacillus* | *reuteri* | [46] | |
| tryptophan | Bacillota | Clostridia | Clostridiales | | Lachnospiraceae | *Blautia* | *coccoides* | [47] | |
| tryptophan | Pseudomonadota | Betaproteobacteria | Burkholderiales | | Burkholderiaceae | *Burkholderia* | (Genus level) | [47] | |

BSH, bile salt hydrolase; TMA, Trimethylamine; SCFAs, Short-chain fatty acids

**Supplementary Table 2. The alteration in gut microbiota during hepatocellular carcinoma inhibition by small molecules or traditional Chinese medicine**

| Substance Name | Increased Key Microbiota Taxa | Decreased Key Microbiota Taxa | Reference |
| --- | --- | --- | --- |
| Jianpi-Huatan-Huoxue-Anshen formula | Actinobacteria, AF12, Adlercreutzia, Clostridium, Coriobacteriaceae Paraprevotella, Lactobacillus | Mucipirillum, Odoribacter, RF32, YS2, Rikenellaceae, p_Deferribacteres | [48] |
| Ulva lactuca ulvan | Agathobacter, Ruminiclostridium, Lactobacillus | Tenericutes, Parabacteroides, Holdemania, unidentified_Clostridiales, | [49] |
| Xierezhuyubuxu decoction | Akkermansia muciniphila, Parabacteroides distasonis | Barnesiella intestinihominis | [50] |
| Astaxanthin | Akkermansia, Faecalibaculum | Colidextribacter, Gemella, Staphylococcus, Anaerotrunces, Enterococcus | [51] |
| Huaier polysaccharides | Akkermansia, Ileibacterium, Bacteroidetes, Limosilactobacillus, Allobaculum, Parabacteroides | Dubosiella, Alloprevotella, Parasutterella | [52] |
| dextran-carbenoxolone | Akkermansia, Lachnospiraceae, Lactobacillus | Rikenellaceae, Escherichia‐Shigella, Helicobacter | [53] |
| Sini Powder | Alistipes, Prevotella | Lachnospira | [54] |
| Safflower yellow | Bacilli, Alphaproteobacteria | Bacteroides, Ersipelotrichaceae incertae sedis | [55] |
| 2,5-dimethylcelecoxib | Bacteroides acidifaciens, Odoribacter laneus, Odoribacter splanchnicus | N/A | [56] |
| Xiayuxue decoction | Bacteroides, Prevotella, Lactobacillus | Firmicutes, Eubacterium, Eubacterium | [57] |
| Recombinant Phycoerythrin | Bacteroidetes, Bacteroides, Alloprevotella, Alistipes, Ruminococcus | Firmicutes, Prevotella, Barnesiella | [58] |
| Pholiota adiposa | Bacteroidetes, Firmicutes, Lactobacillus, Bacteroidales_S24 − 7_group_norank, Bacteroidales, Alloprevotella, Alistipes | Proteobacteria, Lachnospiraceae_NK4A136_group, Prevotellaceae_UCG − 001, Helicobacter, Prevotellaceae_UCG − 003 | [59] |
| Ginsenoside Rk3 | Bacteroidetes, Lachnospiraceae, Bifidobacteriaceae, Akkermansia, Lactobacillus, Oscillibacter, Bifidobacterium | Firmicutes, Ruminococcaceae, Helicobacte | [60] |
| Ginseng glucosyl oleanolate | Bacteroidetes, Lactobacillus, Bacteroides, Clostridium, Enterococcus | Firmicutes | [61] |
| Nanoparticle Conjugation of Ginsenoside Rg3 | Bacteroidetes, Verrucomicrobia, Ruminococcaceae, Barnesiella, Bacteroides, Akkermansia, Helicobacteraceae | Firmicutes, Lachnospiraceae, Rikenella, Clostridiales, | [62] |
| Curcumae | Bifidobacterium, Lactobacillus | Mucispirillum, Bacteroides, Parabacteroides, Clostridium | [63] |
| Nimbolide | Bifidobacterium, Lactobacillus | Escherichia coli, Enterococcus, Bacteroide | [64] |
| Brassica rapa L. polysaccharides | Clostridium, Lachnospira | Bacteroidia, Alistipes, Rikenellaceae | [65] |
| Panax ginseng | Coprococcus, Oscillospira, Anaerotruncus, Ruminococcus, Streptococcus, Roseburia | Prevotella, Bacteroides, | [66] |
| Shaoyao Ruangan Mixture | Desulfovibrio, Turicibacter, Clostridium | Alloprevotella, Bacteroides | [67] |
| THSWD | Duncaniella (and its metabolite Glabrol), Odoribacter, Parabacteroides Rikenellaceae_RC9_gut_group, Bacteroidales_unclassified, Enterococcus, Acinetobacter, Alloprevotella, and Morganella | Anaeroplasma, Muribacter, Aerococcus, Tannerellaceae_unclassified, Corynebacterium, Bergeyella, Fusobacterium | [68] |
| Ganfule capsule | Firmicute, Lactobacillus, Bacilli, Lactobacillales,Lactobacillus,Lactobacillaceae,Firmicutes, Lactobaccillus_reuteri and Lactobacciluls_gasseri | Proteobacteria, Bacteroidetes, Actinobacteria, Patescibacteria, Caulobacter, Desulfovibrio, Candidatus_Arthromitus, Enterorhabdus, Candidatus_Saccharimonas, Staphylococcus, Lachnospiraceae_NK4A136_group , Corynebacterium | [69] |
| Quercetin | Firmicutes, Actinobacteria, Verrucomicrobiota, Dubosiella, Akkermansia | N/A | [70] |
| Tomato Powder | Firmicutes, Bacteroides, Lactobacillus, Bifidobacterium, Clostridium sp. Clone-9 | Bacteroidetes, Deferribacteres, Bacteroides, Mucispirillum, Clostridium, Parabacteroides, Mucispirillum schaedleri, Clostridium sp.ID4 | [71] |
| Stigmasterol | Lactobacillus johnsonii, Lactobacillus murinus, and Lactobacillus reuteri | N/A | [72] |
| nanovesicles from Phellinus linteus | Lactobacillus, Turicibacter, Enterorhabdus | Alloprevotella, Muribaculaceae, Butyricimonas | [73] |
| Morus nigra L. leaves extracted nanoparticles | Lactobacillus, Turicibacter, Firmicutes | Escherichia-Shigella, Uncultured bacterium Prevotellaceae NK3B31 group, Uncultured bacterium Prevotellaceae UCG-001, Bacteroidota, Patescibacteria | [74] |
| Selenium-rich royal jelly | Muribaculaceae | Rikenellaceae, Prevotellaceae, Ruminococcaceae | [75] |
| Atorvastatin | N/A | Mucispirillum schaedleri_Otu038, Desulfovibrio_Otu047, Anaerotruncus_Otu107, Desulfovibrionaceae_Otu073 | [76] |
| Celastrol | N/A | Bacteroides fragilis | [77] |
| Jiedu granule | N/A | Clostridium XI, Peptostre-ptococcaceae | [78] |
| Grifola frondosa Polysaccharide-protein | norank_f__Muribaculaceae, Bacillus | Lactobacillus | [79] |
| Berberine | norank_f_Muribaculaceae, Bacteroides, Lachnospiraceae_NK4A136_group, Rikenellaceae_RC9_gut_group, Alistipes, unclassified_f_Lachnospiraceae, Parabacteroides, norank_f_Lachnospiraceae, Blautia, Colidextribacter, Lachnoclostridium, Oscillibacter, norank_f_Ruminococcaeae, Rikenella, Roseburia, unclassified_f_ Ruminococcaeae | N/A | [80] |
| Echinacea purpurea polysaccharide | Propionic acid and butyric acid-producing gut microbiota such as Coprococcus, Clostridium and Roseburia | N/A | [81] |
| Xiayuxue Decoction | Spirochaetota, Prevotellaceae, Lachnospiraceae, Spirochaetaceae, Lactobacillaceae, Oscillospiraceae, Treponema, Prevotella_9, Lactobacillus, Prevotellaceae_NK3B31_group | Proteobacteria, Erysipelotrichaceae, Enterobacteriaceae, Bacteroides, Escherichia-Shigella, Dubosiella | [82] |

[1] Zha A, Qi M, Deng Y, Li H, Wang N, et al. (2024) Gut Bifidobacterium pseudocatenulatum protects against fat deposition by enhancing secondary bile acid biosynthesis. Imeta 3(6): e261.

[2] Han L, Pendleton A, Singh A, Xu R, Scott SA, et al. (2025) Chemoproteomic profiling of substrate specificity in gut microbiota-associated bile salt hydrolases. Cell Chem Biol 32(1): 145-156.e149.

[3] Kaewarsar E, Chaiyasut C, Lailerd N, Makhamrueang N, Peerajan S, et al. (2023) Effects of Synbiotic Lacticaseibacillus paracasei, Bifidobacterium breve, and Prebiotics on the Growth Stimulation of Beneficial Gut Microbiota. Foods 12(20).

[4] Yang Z, Lin Z, You Y, Zhang M, Gao N, et al. (2025) Gut Microbiota-Derived Hyocholic Acid Enhances Type 3 Immunity and Protects Against Salmonella enterica Serovar Typhimurium in Neonatal Rats. Adv Sci (Weinh) 12(10): e2412071.

[5] Sun X, Chen Z, Yu L, Zeng W, Sun B, et al. (2023) Bacteroides dorei BDX-01 alleviates DSS-induced experimental colitis in mice by regulating intestinal bile salt hydrolase activity and the FXR-NLRP3 signaling pathway. Front Pharmacol 14: 1205323.

[6] Jia F, Du L, He J, Zhang Z, Hou X, et al. (2024) Tong-Xie-Yao-Fang strengthens intestinal feedback control of bile acid synthesis to ameliorate irritable bowel syndrome by enhancing bile salt hydrolase-expressing microbiota. J Ethnopharmacol 331: 118256.

[7] Chen Z, Chen H, Huang W, Guo X, Yu L, et al. (2024) Bacteroides fragilis alleviates necrotizing enterocolitis through restoring bile acid metabolism balance using bile salt hydrolase and inhibiting FXR-NLRP3 signaling pathway. Gut Microbes 16(1): 2379566.

[8] Luo Y, Li M, Wang T, Zhou NN, Qiao F, et al. (2023) Bacillus cereus Alters Bile Acid Composition and Alleviates High-Carbohydrate Diet-Induced Hepatic Lipid Accumulation in Nile Tilapia (Oreochromis niloticus). J Agric Food Chem 71(12): 4825-4836.

[9] Song Z, Feng S, Zhou X, Song Z, Li J, et al. (2023) Taxonomic identification of bile salt hydrolase-encoding lactobacilli: Modulation of the enterohepatic bile acid profile. Imeta 2(3): e128.

[10] Hu S, Tang B, Lu C, Wang S, Wu L, et al. (2024) Lactobacillus rhamnosus GG ameliorates triptolide-induced liver injury through modulation of the bile acid-FXR axis. Pharmacol Res 206: 107275.

[11] Foley MH, O'Flaherty S, Allen G, Rivera AJ, Stewart AK, et al. (2021) Lactobacillus bile salt hydrolase substrate specificity governs bacterial fitness and host colonization. Proc Natl Acad Sci U S A 118(6).

[12] Li X, Xiao Y, Huang Y, Song L, Li M, et al. (2022) Lactobacillus gasseri RW2014 Ameliorates Hyperlipidemia by Modulating Bile Acid Metabolism and Gut Microbiota Composition in Rats. Nutrients 14(23).

[13] Xu J, Zhou Y, Cheng S, Zhao Y, Yan J, et al. (2023) Lactobacillus johnsonii Attenuates Liver Steatosis and Bile Acid Dysregulation in Parenteral Nutrition-Fed Rats. Metabolites 13(10).

[14] Jones ML, Martoni CJ, Prakash S (2012) Cholesterol lowering and inhibition of sterol absorption by Lactobacillus reuteri NCIMB 30242: a randomized controlled trial. Eur J Clin Nutr 66(11): 1234-1241.

[15] Feng J, Ma H, Yue Y, Wang L, Hao K, et al. (2023) Saikosaponin a ameliorates diet-induced fatty liver via regulating intestinal microbiota and bile acid profile in laying hens. Poult Sci 102(12): 103155.

[16] He G, Zhang B, Yi K, Chen T, Shen C, et al. (2024) Heat stress-induced dysbiosis of the gut microbiota impairs spermatogenesis by regulating secondary bile acid metabolism in the gut. Sci Total Environ 937: 173305.

[17] Jalil A, Perino A, Dong Y, Imbach J, Volet C, et al. (2025) Bile acid 7α-dehydroxylating bacteria accelerate injury-induced mucosal healing in the colon. EMBO Mol Med 17(5): 889-908.

[18] Hisatomi A, Kastawa N, Song I, Ohkuma M, Fukiya S, et al. (2023) Claveliimonas bilis gen. nov., sp. nov., deoxycholic acid-producing bacteria isolated from human faeces, and reclassification of Sellimonas monacensis Zenner et al. 2021 as Claveliimonas monacensis comb. nov. Int J Syst Evol Microbiol 73(9).

[19] Song I, Gotoh Y, Ogura Y, Hayashi T, Fukiya S, et al. (2021) Comparative Genomic and Physiological Analysis against Clostridium scindens Reveals Eubacterium sp. c-25 as an Atypical Deoxycholic Acid Producer of the Human Gut Microbiota. Microorganisms 9(11).

[20] Kitahara M, Takamine F, Imamura T, Benno Y (2001) Clostridium hiranonis sp. nov., a human intestinal bacterium with bile acid 7alpha-dehydroxylating activity. Int J Syst Evol Microbiol 51(Pt 1): 39-44.

[21] Wells JE, Hylemon PB (2000) Identification and characterization of a bile acid 7alpha-dehydroxylation operon in Clostridium sp. strain TO-931, a highly active 7alpha-dehydroxylating strain isolated from human feces. Appl Environ Microbiol 66(3): 1107-1113.

[22] (2024) Puerarin alleviates atherosclerosis via the inhibition of Prevotella copri and its trimethylamine production. Gut 73(12): 1934-1943.

[23] Rath S, Heidrich B, Pieper DH, Vital M (2017) Uncovering the trimethylamine-producing bacteria of the human gut microbiota. Microbiome 5(1): 54.

[24] Wu WK, Chen CC, Liu PY, Panyod S, Liao BY, et al. (2019) Identification of TMAO-producer phenotype and host-diet-gut dysbiosis by carnitine challenge test in human and germ-free mice. Gut 68(8): 1439-1449.

[25] Wang Z, Roberts AB, Buffa JA, Levison BS, Zhu W, et al. (2015) Non-lethal Inhibition of Gut Microbial Trimethylamine Production for the Treatment of Atherosclerosis. Cell 163(7): 1585-1595.

[26] Wang Q, Guo M, Liu Y, Xu M, Shi L, et al. (2022) Bifidobacterium breve and Bifidobacterium longum Attenuate Choline-Induced Plasma Trimethylamine N-Oxide Production by Modulating Gut Microbiota in Mice. Nutrients 14(6).

[27] Kashyap J, Ringiesn JR, Schwab N, Ferguson DJ, Jr. (2022) Isolation and characterization of a novel choline degrading Citrobacter amalonaticus strain from the human gut. Curr Res Microb Sci 3: 100157.

[28] Dalla Via A, Gargari G, Taverniti V, Rondini G, Velardi I, et al. (2019) Urinary TMAO Levels Are Associated with the Taxonomic Composition of the Gut Microbiota and with the Choline TMA-Lyase Gene (cutC) Harbored by Enterobacteriaceae. Nutrients 12(1).

[29] Jameson E, Doxey AC, Airs R, Purdy KJ, Murrell JC, et al. (2016) Metagenomic data-mining reveals contrasting microbial populations responsible for trimethylamine formation in human gut and marine ecosystems. Microb Genom 2(9): e000080.

[30] Massmig M, Reijerse E, Krausze J, Laurich C, Lubitz W, et al. (2020) Carnitine metabolism in the human gut: characterization of the two-component carnitine monooxygenase CntAB from Acinetobacter baumannii. J Biol Chem 295(37): 13065-13078.

[31] Smith PM, Howitt MR, Panikov N, Michaud M, Gallini CA, et al. (2013) The microbial metabolites, short-chain fatty acids, regulate colonic Treg cell homeostasis. Science 341(6145): 569-573.

[32] Hu C, Xu B, Wang X, Wan WH, Lu J, et al. (2023) Gut microbiota-derived short-chain fatty acids regulate group 3 innate lymphoid cells in HCC. Hepatology 77(1): 48-64.

[33] Lau HC, Zhang X, Ji F, Lin Y, Liang W, et al. (2024) Lactobacillus acidophilus suppresses non-alcoholic fatty liver disease-associated hepatocellular carcinoma through producing valeric acid. EBioMedicine 100: 104952.

[34] Kumar M, Kaur R, Kanthaje S, Dhiman RK, Chakraborti A (2023) Bacterial metabolite butyrate in modulating sorafenib-targeted microRNAs to curtail its resistance in hepatocellular carcinoma. J Cancer Res Clin Oncol 149(9): 5823-5839.

[35] Luo M, Du Y, Liu X, Zhang S, Zhu W, et al. (2025) Fecal microbiota transplantation alleviates cirrhotic portal hypertension in rats via butyrate-mediated HDAC3 inhibition and PI3K/Akt/eNOS signaling regulation. Eur J Pharmacol 1002: 177781.

[36] Lee J, d'Aigle J, Atadja L, Quaicoe V, Honarpisheh P, et al. (2020) Gut Microbiota-Derived Short-Chain Fatty Acids Promote Poststroke Recovery in Aged Mice. Circ Res 127(4): 453-465.

[37] Verma S, Dutta SK, Firnberg E, Phillips L, Vinayek R, et al. (2021) Identification and engraftment of new bacterial strains by shotgun metagenomic sequence analysis in patients with recurrent Clostridioides difficile infection before and after fecal microbiota transplantation and in healthy human subjects. PLoS One 16(7): e0251590.

[38] Song Q, Zhang X, Liu W, Wei H, Liang W, et al. (2023) Bifidobacterium pseudolongum-generated acetate suppresses non-alcoholic fatty liver disease-associated hepatocellular carcinoma. J Hepatol 79(6): 1352-1365.

[39] Xia J, Lv L, Liu B, Wang S, Zhang S, et al. (2022) Akkermansia muciniphila Ameliorates Acetaminophen-Induced Liver Injury by Regulating Gut Microbial Composition and Metabolism. Microbiol Spectr 10(1): e0159621.

[40] Miao C, Wang L, Wang H, Shen Y, Man C, et al. (2024) Lacticaseibacillus plantarum postbiotics prepared by the combined technique of pasteurization and ultrasound: effective measures to alleviate obesity based on the SCFAs-GPR41/GPR43 signaling pathway. Food Funct 15(22): 11005-11019.

[41] Gao P, Nie Y, Zhao L, Zhang J, Ge W (2025) Lactococcus lactis Subsp. lactis LL-1 and Lacticaseibacillus paracasei LP-16 Influence the Gut Microbiota and Metabolites for Anti-Obesity and Hypolipidemic Effects in Mice. Antioxidants (Basel) 14(5).

[42] Yoon SJ, Yu JS, Min BH, Gupta H, Won SM, et al. (2023) Bifidobacterium-derived short-chain fatty acids and indole compounds attenuate nonalcoholic fatty liver disease by modulating gut-liver axis. Front Microbiol 14: 1129904.

[43] Min BH, Devi S, Kwon GH, Gupta H, Jeong JJ, et al. (2024) Gut microbiota-derived indole compounds attenuate metabolic dysfunction-associated steatotic liver disease by improving fat metabolism and inflammation. Gut Microbes 16(1): 2307568.

[44] Sun Y, Liu X, Zhao L, Li D, Guan K, et al. (2025) Lacticaseibacillus rhamnosus HF01 postbiotics reprogram gut microbial tryptophan metabolism to coordinate enterohepatic barrier-insulin signaling axis. Curr Res Food Sci 11: 101111.

[45] Guo Z, Yang S, Qi L, Ma X, Wang Y, et al. (2025) Lactobacillus acidophilus KLDS1.0901 ameliorates non-alcoholic fatty liver disease by modulating the tryptophan metabolite indole-3-aldehyde and acting on its receptor AhR. Food Funct 16(12): 4939-4957.

[46] Chi YY, Xiang JY, Li HM, Shi HY, Ning K, et al. (2024) Schisandra chinensis polysaccharide prevents alcohol-associated liver disease in mice by modulating the gut microbiota-tryptophan metabolism-AHR pathway axis. Int J Biol Macromol 282(Pt 2): 136843.

[47] Niu Y, Hu X, Song Y, Wang C, Luo P, et al. (2024) Blautia Coccoides is a Newly Identified Bacterium Increased by Leucine Deprivation and has a Novel Function in Improving Metabolic Disorders. Adv Sci (Weinh) 11(18): e2309255.

[48] Wang YN, Zhai XY, Wang Z, Gao CL, Mi SC, et al. (2024) Jianpi-Huatan-Huoxue-Anshen formula ameliorates gastrointestinal inflammation and microecological imbalance in chemotherapy-treated mice transplanted with H22 hepatocellular carcinoma. World journal of gastrointestinal oncology 16(10): 4209-4231.

[49] Qiu Y, Xu J, Liao W, Wen Y, Jiang S, et al. (2023) Suppression of hepatocellular carcinoma by Ulva lactuca ulvan via gut microbiota and metabolite interactions. Journal of advanced research 52: 103-117.

[50] Zeng Z, Feng M, He F, Zhang E, Li X, et al. (2024) Gut microbiota mediates the pro-pyroptosis effect of xierezhuyubuxu decoction in hepatocellular carcinoma. Front Microbiol 15: 1481111.

[51] Ren P, Yu X, Yue H, Tang Q, Wang Y, et al. (2023) Dietary supplementation with astaxanthin enhances anti-tumor immune response and aids the enhancement of molecularly targeted therapy for hepatocellular carcinoma. Food & function 14(18): 8309-8320.

[52] Li X, Zhang H, Deng Y, Fang Q, Zhang X, et al. (2025) Huaier polysaccharides inhibits hepatocellular carcinoma via gut microbiota mediated M2 macrophage polarization. International journal of biological macromolecules 293: 139357.

[53] Yao H, Ma S, Huang J, Si X, Yang M, et al. (2024) Trojan-Horse Strategy Targeting the Gut-Liver Axis Modulates Gut Microbiome and Reshapes Microenvironment for Orthotopic Hepatocellular Carcinoma Therapy. Advanced science (Weinheim, Baden-Wurttemberg, Germany) 11(44): e2310002.

[54] Mei S, Deng Z, Meng FY, Guo QQ, Tao HY, et al. (2025) Sini Powder Alleviates Stress Response and Suppresses Hepatocellular Carcinoma Development by Restoring Gut Microbiota. Chinese journal of integrative medicine.

[55] Fu H, Liu X, Jin L, Lang J, Hu Z, et al. (2021) Safflower yellow reduces DEN-induced hepatocellular carcinoma by enhancing liver immune infiltration through promotion of collagen degradation and modulation of gut microbiota. Food & function 12(21): 10632-10643.

[56] Pan B, Chen Z, Zhang X, Wang Z, Yao Y, et al. (2023) 2,5-dimethylcelecoxib alleviated NK and T-cell exhaustion in hepatocellular carcinoma via the gastrointestinal microbiota-AMPK-mTOR axis. J Immunother Cancer 11(6).

[57] Deng Z, Ouyang Z, Mei S, Zhang X, Li Q, et al. (2024) Enhancing NKT cell-mediated immunity against hepatocellular carcinoma: Role of XYXD in promoting primary bile acid synthesis and improving gut microbiota. Journal of ethnopharmacology 318(Pt B): 116945.

[58] Qi H, Liu Y, Qi X, Liang H, Chen H, et al. (2019) Dietary Recombinant Phycoerythrin Modulates the Gut Microbiota of H22 Tumor-Bearing Mice. Marine drugs 17(12).

[59] Wang XY, Zhang Y, Liu FF (2022) Influence of Pholiota adiposa on gut microbiota and promote tumor cell apoptosis properties in H22 tumor-bearing mice. Scientific reports 12(1): 8589.

[60] Qu L, Ma X, Fan D (2021) Ginsenoside Rk3 Suppresses Hepatocellular Carcinoma Development through Targeting the Gut-Liver Axis. Journal of agricultural and food chemistry 69(35): 10121-10137.

[61] Ai Z, Liu S, Zhang J, Hu Y, Tang P, et al. (2024) Ginseng Glucosyl Oleanolate from Ginsenoside Ro, Exhibited Anti-Liver Cancer Activities via MAPKs and Gut Microbiota In Vitro/Vivo. Journal of agricultural and food chemistry 72(14): 7845-7860.

[62] Ren Z, Chen X, Hong L, Zhao X, Cui G, et al. (2020) Nanoparticle Conjugation of Ginsenoside Rg3 Inhibits Hepatocellular Carcinoma Development and Metastasis. Small (Weinheim an der Bergstrasse, Germany) 16(2): e1905233.

[63] Zhang Y, Li X, Li X (2021) Curcumae Ameliorates Diethylnitrosamine-Induced Hepatocellular Carcinoma via Alteration of Oxidative Stress, Inflammation and Gut Microbiota. Journal of inflammation research 14: 5551-5566.

[64] Ram AK, Vairappan B, Srinivas BH (2022) Nimbolide attenuates gut dysbiosis and prevents bacterial translocation by improving intestinal barrier integrity and ameliorating inflammation in hepatocellular carcinoma. Phytotherapy research : PTR 36(5): 2143-2160.

[65] Kong H, Yang J, Wang X, Mamat N, Xie G, et al. (2024) The combination of Brassica rapa L. polysaccharides and cisplatin enhances the anti liver cancer effect and improves intestinal microbiota and metabolic disorders. Int J Biol Macromol 265(Pt 1): 130706.

[66] Hou Z, Song F, Xing J, Zheng Z, Liu S, et al. (2022) Comprehensive fecal metabolomics and gut microbiota for the evaluation of the mechanism of Panax Ginseng in the treatment of Qi-deficiency liver cancer. Journal of ethnopharmacology 292: 115222.

[67] Zhen H, Qian X, Fu X, Chen Z, Zhang A, et al. (2019) Regulation of Shaoyao Ruangan Mixture on Intestinal Flora in Mice With Primary Liver Cancer. Integrative cancer therapies 18: 1534735419843178.

[68] Zhu Z, Zuo S, Zhu Z, Wang C, Du Y, et al. (2025) THSWD upregulates the LTF/AMPK/mTOR/Becn1 axis and promotes lysosomal autophagy in hepatocellular carcinoma cells by regulating gut flora and metabolic reprogramming. International immunopharmacology 148: 114091.

[69] Xu F, Li H, Pan Y, Zeng Y, Li J, et al. (2022) Effects of Ganfule capsule on microbial and metabolic profiles in anti-hepatocellular carcinoma. Journal of applied microbiology 132(3): 2280-2292.

[70] Wu R, Xiong J, Zhou T, Zhang Z, Huang Z, et al. (2023) Quercetin/Anti-PD-1 Antibody Combination Therapy Regulates the Gut Microbiota, Impacts Macrophage Immunity and Reshapes the Hepatocellular Carcinoma Tumor Microenvironment. Frontiers in bioscience (Landmark edition) 28(12): 327.

[71] Xia H, Liu C, Li CC, Fu M, Takahashi S, et al. (2018) Dietary Tomato Powder Inhibits High-Fat Diet-Promoted Hepatocellular Carcinoma with Alteration of Gut Microbiota in Mice Lacking Carotenoid Cleavage Enzymes. Cancer prevention research (Philadelphia, Pa) 11(12): 797-810.

[72] Huo R, Yang WJ, Liu Y, Liu T, Li T, et al. (2024) Stigmasterol: Remodeling gut microbiota and suppressing tumor growth through Treg and CD8+ T cells in hepatocellular carcinoma. Phytomedicine : international journal of phytotherapy and phytopharmacology 129: 155225.

[73] Zu M, Liu G, Chen N, Chen L, Gao Q, et al. (2024) Oral exosome-like nanovesicles from Phellinus linteus suppress metastatic hepatocellular carcinoma by reactive oxygen species generation and microbiota rebalancing. Nanoscale 16(16): 8046-8059.

[74] Gao Q, Chen N, Li B, Zu M, Ma Y, et al. (2024) Natural lipid nanoparticles extracted from Morus nigra L. leaves for targeted treatment of hepatocellular carcinoma via the oral route. Journal of nanobiotechnology 22(1): 4.

[75] Chi X, Liu Z, Wei W, Hu X, Wang Y, et al. (2021) Selenium-rich royal jelly inhibits hepatocellular carcinoma through PI3K/AKT and VEGF pathways in H22 tumor-bearing mice. Food & function 12(19): 9111-9127.

[76] Zhang X, Coker OO, Chu ES, Fu K, Lau HCH, et al. (2021) Dietary cholesterol drives fatty liver-associated liver cancer by modulating gut microbiota and metabolites. Gut 70(4): 761-774.

[77] Zeng D, Zhang L, Luo Q (2023) Celastrol-regulated gut microbiota and bile acid metabolism alleviate hepatocellular carcinoma proliferation by regulating the interaction between FXR and RXRα in vivo and in vitro. Front Pharmacol 14: 1124240.

[78] Yifu F, Hetong Z, Yani Z, Zifei Y, Juan DU, et al. (2022) Effectiveness of Jiedu granule on gut microbiota in patients with advanced hepatocellular carcinoma: a randomized controlled trial. Journal of traditional Chinese medicine = Chung i tsa chih ying wen pan 42(6): 988-996.

[79] Zhao J, He R, Zhong H, Liu S, Liu X, et al. (2023) A cold-water extracted polysaccharide-protein complex from Grifola frondosa exhibited anti-tumor activity via TLR4-NF-κB signaling activation and gut microbiota modification in H22 tumor-bearing mice. International journal of biological macromolecules 239: 124291.

[80] Shou JW, Shaw PC (2023) Berberine activates PPARδ and promotes gut microbiota-derived butyric acid to suppress hepatocellular carcinoma. Phytomedicine : international journal of phytotherapy and phytopharmacology 115: 154842.

[81] Jing G, Xu W, Ma W, Yu Q, Zhu H, et al. (2024) Echinacea purpurea polysaccharide intervene in hepatocellular carcinoma via modulation of gut microbiota to inhibit TLR4/NF-κB pathway. International journal of biological macromolecules 261(Pt 2): 129917.

[82] Zhou T, Pei L, Chen Y, Wang X, Fang H, et al. (2025) Xiayuxue Decoction Plays an Antidevelopment Role in Hepatocellular Carcinoma through Intestinal-Hepatic Axis. Digestion: 1-15.
